# Can Oxygen Isotopes in Tree Rings Be Used to Detect Stomatal Responses to Global Change?

**DOI:** 10.1111/gcb.70604

**Published:** 2025-11-15

**Authors:** Imogen Carter, Roel Brienen, Manuel Gloor

**Affiliations:** ^1^ School of Geography University of Leeds Leeds UK

**Keywords:** Craig‐Gordon‐Dongmann model, dendrochronology, dual‐isotope approach, intrinsic water‐use efficiency, Péclet effect, stable isotope ratio, stomatal conductance

## Abstract

Stomatal conductance (g_s_) regulates CO_2_ and water fluxes of plants. Although experiments have shown that g_s_ decreases with elevated CO_2_, it is unclear how g_s_ is responding in situ to long‐term exposures to rising CO_2_ and a changing climate. Tree ring isotope analysis provides a unique method to assess tree ecophysiological responses to long‐term exposures of slowly changing environmental conditions. In particular, it has been suggested that changes in g_s_ can potentially be inferred from tree ring stable oxygen isotope ratios (δ^18^O_trc_). Several studies have indeed used δ^18^O_trc_ trends to conclude that g_s_ has not significantly changed from pre‐industrial values. However, it remains unclear whether δ^18^O_trc_ is sufficiently sensitive to detect the magnitude of change in g_s_ expected due to CO_2_ increases and climatic changes. Here, we evaluate the sensitivity of δ^18^O_trc_ trends to CO_2_ and climate induced changes in g_s_, and to VPD and temperature increases since the beginning of the 20th century, using current theoretical models. We find that temporal changes in g_s_ only significantly affect δ^18^O_trc_ trends when the Péclet effect is present, and then only in dry climates. In contrast to the weak effects of g_s_ on δ^18^O_trc_ trends, we find that temporal increases in VPD and temperature, independent of changes in g_s_, have far greater contributions to δ^18^O_trc_ trends. Thus, this increasingly popular method should be used with caution, because it is highly challenging to unambiguously attribute trends in δ^18^O_trc_ to changes in g_s_. Despite current limitations, we recommend how future studies can address these challenges in efforts to detect long‐term g_s_ trends from tree ring records.

## Graphical abstract

We model changes in tree stomatal conductance (g_s_) due to increases in CO_2_ and changes in climate since the beginning of the 20th century, and subsequent changes in tree ring oxygen isotopes. We analyse how well long‐term g_s_ changes are reflected in tree ring oxygen isotope trends for different physiological and climatic assumptions. We find that long‐term tree ring oxygen isotope trends cannot be unambiguously attributed to changes in g_s_ caused by CO_2_ and climatic changes.

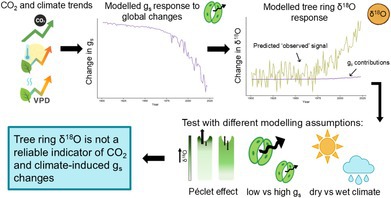

## 1 Introduction

Plants are changing their functioning in response to rising atmospheric CO_2_ concentrations and climatic changes, modulating the vegetation–climate system (Li 2024). Of particular interest are the ecophysiological responses of trees and forests to global change, as forests cover about one third of Earth's land surface, and account for approximately 91% of the global land carbon sink (Pan et al. 2024; FAO 2020). Forests also play a significant role in the hydrological cycle, for example by mediating atmospheric water transport over land, regulating surface water runoff and recycling 40% of land‐based precipitation via transpiration (Van Der Ent et al. 2010; Ellison et al. 2017). Free Air CO_2_ Enrichment (FACE) studies (Bader et al. 2013; Norby and Zak 2011; Norby et al. 2024; Walker et al. 2019), eddy covariance flux measurements (Keenan et al. 2013; Fernández‐Martínez et al. 2017), and tree ring stable carbon isotope ratio (δ^13^C) studies (Saurer et al. 2004; Frank et al. 2015; Van Der Sleen et al. 2015) indicate physiological changes in trees in response to rising CO_2_ levels and climatic changes. Stomata regulate the fluxes of CO_2_ into, and water out of, the leaf, and thus, play a key role in these ecophysiological changes. Consequently, it is important to understand how stomata are responding to global change.

In controlled experiments, and rapid step increases in CO_2_ concentrations (e.g., FACE studies), it is generally observed that stomatal conductance (g_s_) decreases with rising CO_2_ levels (Ainsworth and Rogers 2007; Gardner et al. 2023; Liang et al. 2023; Lammertsma et al. 2011; Medlyn et al. 2001). Stomatal responses are further modulated by rising atmospheric temperatures depending on water availability (Liang et al. 2023; Urban et al. 2017). Yet, questions remain as to what degree these experiments are representative of real‐time responses of trees to global change. Thus, there remains a need for in situ evidence of stomatal responses to long‐term exposures to increasing CO_2_ and climatic changes.

The stable oxygen isotope composition (δ^18^O) of leaf water is controlled in part by g_s_, and thus changes in leaf δ^18^O over time can reflect changes in g_s_ (Barbour et al. 2000). Leaf water δ^18^O is partially integrated within δ^18^O of tree ring cellulose, δ^18^O_trc_ (Barbour and Farquhar 2000), and changes in g_s_ may thus potentially be recorded in annual tree ring δ^18^O. Therefore, if g_s_ trends can be reliably inferred from δ^18^O_trc_ signals, tree ring δ^18^O analysis would be suitable to estimate the magnitude of in situ past stomatal responses of trees to long‐term exposures to increasing CO_2_ and climatic changes.

According to Farquhar et al. (1982), δ^13^C of leaf and cambial cellulose is related to the ratio of photosynthetic rate (A) to g_s_, which is termed intrinsic water use efficiency (iWUE). This ratio reflects the balance between the fluxes of carbon and water, with A related to primary production, and g_s_ regulating the rate of transpiration and CO_2_ flux into the leaf intercellular space (Osmond et al. 1980). The majority of tree ring δ^13^C records indicate sustained increases in iWUE, and this has been attributed to rising CO_2_ levels (Frank et al. 2015; Saurer et al. 2004; Van Der Sleen et al. 2015). To the extent that iWUE records and the Farquhar model can be trusted, and thus, that A/g_s_ is increasing, it remains unclear whether this trend is caused by changes in A, g_s_ or both.

Scheidegger et al. (2000) proposed the ‘dual‐isotope approach’ to constrain plant δ^13^C‐derived changes in iWUE by using plant δ^18^O to infer changes in g_s_. This approach seeks to take advantage of independent information of A and g_s_ reflected in plant δ^18^O and δ^13^C records to determine the relative contributions of A and g_s_ to iWUE trends. This is based on the assumption that g_s_ and plant δ^18^O are negatively related, when all other variables are held constant. Indeed, several studies report a robust negative relationship between g_s_ and δ^18^O of leaf cellulose in controlled laboratory settings (Barbour et al. 2000; Grams et al. 2007), and in situ (Sullivan and Welker 2007; Moreno‐Gutiérrez et al. 2012). The dual‐isotope approach has since been applied to tree ring studies, using δ^18^O of tree ring cellulose to interpret iWUE trends (Nock et al. 2011; Fajardo et al. 2019; Siegwolf et al. 2023). For a more detailed description of the dual‐isotope approach, see the paper of Siegwolf et al. (2023), which reviews more than 250 applications of the dual‐isotope approach in plant physiological studies.

Two recent studies (Guerrieri et al. 2019; Mathias and Thomas 2021) have expanded on the tree ring dual‐isotope approach by back‐calculating ^18^O‐enrichment of leaf water above source water (Δ^18^O_lw_), using δ^18^O_trc_ measurements and estimates for historical variation of source water δ^18^O (δ^18^O_sw_). According to these studies, iWUE increases are largely due to increases in A, as they find that Δ^18^O_lw_, and thus g_s_, remained effectively unchanged. Mathias and Thomas (2021) conclude that g_s_ has not changed in trees at 83% of the study sites, while Guerrieri et al. (2019) found that δ^18^O_trc_ increased in xeric sites, but did not change in trees from mesic sites. This led the authors to conclude that g_s_ reductions were restricted to species in moisture‐limited conditions. However, the methods employed in these studies were criticised on several grounds by Lin et al. (2022). First, longer‐term temporal δ^18^O_sw_ trends at each site were calculated using a spatially explicit model of precipitation δ^18^O, which poorly estimated precipitation δ^18^O trends across time. Secondly, background noise in δ^18^O signals due to interannual climatic variability was not appropriately considered. This is a significant source of error because this application of the dual‐isotope approach requires other factors that influence δ^18^O (e.g., inter‐annual variability of relative humidity) to be constant, in order to infer that changes in δ^18^O are caused by changes to g_s_ (Roden and Siegwolf 2012). Thirdly, the studies assumed a uniform negative g_s_‐δ^18^O relationship across all species, which relies on the assumption that the Péclet effect is of similar magnitude for all species. However, the Péclet effect may not be the same for all species (Barbour et al. 2021; Song et al. 2013). Considering this, Lin et al. (2022) demonstrate that changes in g_s_ in the order of 0.1 mol m^−2^ s^−1^ are on the limit of detectability (0.3‰) in δ^18^O_trc_ trends when the Péclet effect is absent, and changes in δ^18^O_trc_ may be even smaller if the Péclet effect varies with transpiration rate. This implies that g_s_ trends may not always be detectable in δ^18^O_trc_ signals. Additionally, theory suggests that changes in g_s_ have their strongest effect on δ^18^O in dry conditions (Roden and Siegwolf 2012). Thus, it may be that decreases in g_s_ are simply only detectable in δ^18^O_trc_ series at water‐limited sites, and not in wet sites, potentially leading to false impressions that g_s_ did not change at these mesic sites.

In summary, it remains unclear what conditions are required for changes in g_s_ due to CO_2_ and anthropogenic climate change to be detectable in δ^18^O_trc_ trends. There is emerging experimental research into the sensitivity of cellulose δ^18^O to the effects of rising CO_2_ concentrations on g_s_ for some grass, legume and herb species (Morgner et al. 2024) and for trees e.g., *Pinus mugo* and *Larix decidua* (Streit et al. 2014). Existing research indicates that increasing temperatures and vapour pressure deficit (VPD) significantly increase δ^18^O_trc_ by driving ^18^O‐enrichment at the evaporating site of the leaf for trees, independent of changes to g_s_ (Kahmen et al. 2011; Cheesman and Cernusak 2016; Streit et al. 2014). Thus, it remains important to quantify the relative contributions of temporal changes in climate, and of g_s_ responses to CO_2_ and climatic changes, to δ^18^O_trc_ trends to determine whether changes in g_s_ due to anthropogenic global change can be reliably inferred from δ^18^O_trc_ signals.

The aim of this article is to determine the sensitivity of δ^18^O_trc_ trends to changes in g_s_ due to anthropogenic CO_2_ emissions and associated climatic changes. We disentangle the sensitivity of Δ^18^O_lw_ and δ^18^O_trc_ trends to simultaneous increases in CO_2_ concentrations, atmospheric temperature and VPD over the past 150 years using standard mechanistic models of oxygen isotopic composition in plant tissues. We do this for an idealised mature tree growing in a Northern latitude temperate climate using various physiological and environmental assumptions to determine under what conditions δ^18^O_trc_ trends can be used to detect a change in g_s_. We discuss the implications of our results in view of the conclusions of recent studies that have used δ^18^O_trc_ trends to infer how g_s_ has changed in response to rising CO_2_ levels. Ultimately, we evaluate whether current applications of tree ring δ^18^O analysis can determine how g_s_ has changed since the beginning of the 20th century.

The next section describes the existing theory for tree stable oxygen isotope models. Then, we describe the modelling approach employed to address the aims of this article, followed by the results and discussion sections.

## 2 Theory and Illustration of Mechanisms

**FIGURE 1:**
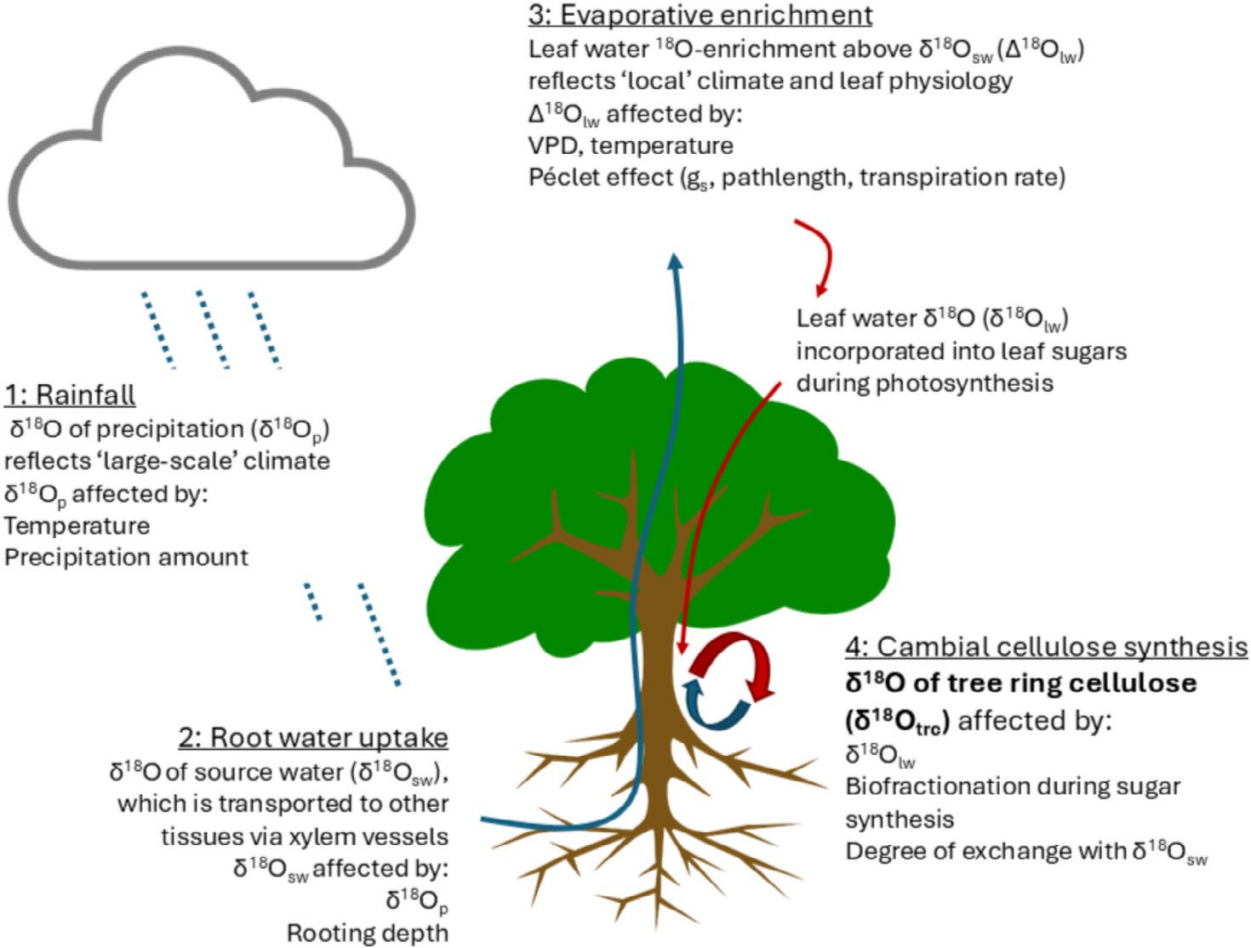
The transfer of oxygen isotope signals from rainwater to tree ring cellulose. δ^18^O of precipitation (1) contains an imprint of climate (temperature, precipitation amount and large‐scale rainout processes). These signals are transferred from precipitation to (2) soil, xylem and leaf water, and then altered by leaf evaporative enrichment (3), biofractionations and exchange with xylem water during cellulose synthesis (4).

According to current understanding, tree ring cellulose δ^18^O (δ^18^O_trc_) is a mixed signal coming from variation in δ^18^O of the source water and evaporative enrichment of that source water signal in the leaf (Figure 1).

The plant source water δ^18^O (δ^18^O_sw_) variation is determined by rainfall δ^18^O, which is controlled by the movement of heavy and light water through the large‐scale hydrological cycle. Rainwater δ^18^O increases with air temperature during condensation, decreases with increasing precipitation intensity, and decreases as the path length of water vapour transport increases (i.e., towards higher latitudes and altitudes, and further inland) due to Rayleigh distillation effects (Dansgaard 1964; Rozanski et al. 1992). Plant source water δ^18^O largely reflects precipitation δ^18^O but this may vary depending on root water uptake from different soil depths. For example, groundwater δ^18^O can vary significantly from precipitation δ^18^O, and kinetic fractionation during evaporation at the soil surface and within the soil pores causes ^18^O‐enrichment of soil water relative to rainwater (Ehleringer and Dawson 1992; Sprenger et al. 2017). Xylem water δ^18^O is equal to δ^18^O_sw_, because no fractionation is assumed during water uptake by roots (Allison et al. 1983; Dawson and Ehleringer 1991).

Water is transported through the tree to the stomatal opening of the leaf via the transpiration stream. The δ^18^O of water at the evaporating site of the leaf (δ^18^O_es_) increases because ‘light’ H_2_^16^O evaporates more readily than ‘heavy’ H_2_^18^O. Enrichment of ^18^O at the evaporating site of the leaf is modelled by the Craig‐Gordon‐Dongmann (CGD) equation (Craig and Gordon 1965; Dongmann et al. 1974; Farquhar and Lloyd 1993):

(1) $$\delta^{18}O_{\mathrm{es}}=\left(\delta^{18}O_{\mathrm{sw}}+\varepsilon_{k}\right)\left(\frac{e_{i}-e_{a}}{e_{i}}\right)+\varepsilon *+\delta^{18}O_{\mathrm{wv}}\left(\frac{e_{a}}{e_{i}}\right)$$

where ε_*k*_ is the kinetic fractionation of water during diffusion through the stomata and leaf boundary layer (‰), *e*_*i*_ is the internal vapour pressure of the leaf at saturation (kPa) (c.f., Appendix S1: Eqn. SI.3.1), *e*_*a*_ is atmospheric vapour pressure (kPa), ε* is the equilibrium fraction between liquid water and vapour during evaporation from the mesophyll (‰), and δ^18^O_wv_ is δ^18^O of atmospheric water vapour (‰).

Kinetic fractionation is typically in the range of 28‰–32‰, and occurs during diffusion of water through the stomata and leaf boundary layer, and is thus related to stomatal conductance (g_s_) and boundary layer conductance (g_b_), according to (Farquhar et al. 1998; Barbour 2007):

(2) $$\varepsilon_{k}=\frac{32{g_{s}}^{-1}+21{g_{b}}^{-1}}{{g_{s}}^{-1}+{g_{b}}^{-1}}$$

Equilibrium fractionation is temperature‐dependent and typically in the range of 9‰–10‰, and occurs during evaporation of water from the site of evaporation in the leaf (Bottinga and Craig 1969):

(3) $$\varepsilon *=2.644-\left(3.206\times \frac{10^{3}}{T_{\text{leaf}}}\;\right)+\left(1.534\times \frac{10^{6}}{{T_{\text{leaf}}}^{2}}\right)$$

where *T*_leaf_ is leaf temperature (Kelvin).

To control for variation in δ^18^O_sw_, ^18^O‐enrichment of water at the evaporating site can be expressed above δ^18^O_sw_ (i.e., Δ^18^O_es_ = δ^18^O_es_ − δ^18^O_sw_), and can be approximated as (Barbour et al. 2004):

(4) $${∆}^{18}O_{\mathrm{es}}\approx \varepsilon *+\varepsilon_{k}\left(1-\frac{e_{a}}{e_{i}}\right)$$

assuming δ^18^O_sw_ = δ^18^O_wv_.

The CGD model shows that Δ^18^O_es_ increases with increasing evaporative demand, i.e., increasing leaf‐to‐air vapour pressure deficit, VPD = (*e*_*i*_ *‐e*_a_), or decreasing relative humidity, RH (e_a_/e_*i*_). Under 100% RH (e_a_/e_*i*_ = 1), the contribution of *ε*_*k*_ to Δ^18^O_es_ is 0, and Δ^18^O_es_ is thus equal to ε*. Under decreasing RH, the contributions of *ε*_*k*_ to Δ^18^O_es_ increase, leading to higher Δ^18^O_es_.

For applications of the CGD model across large temporal and spatial scales (e.g., δ^18^O signals integrated into tree ring material) it is usually assumed that source water and atmospheric water vapour are equal and in steady‐state isotopic equilibrium, i.e., δ^18^O_sw_ = δ^18^O_wv_ (Cernusak et al. 2016). This steady‐state assumption is not necessarily correct, but non‐steady state models are highly complex and do not significantly improve modelling accuracy compared to the steady‐state assumption (Cernusak et al. 2016). Thus, for the purpose of this study we only use the model with this assumption.

The first leaf isotope models assumed that there is no compartmentalisation of water in the leaf, that is, bulk leaf water δ^18^O is the same as δ^18^O at the site of evaporation (i.e., δ^18^O_lw_ = δ^18^O_es_). However, multiple studies have demonstrated that leaf and cellulose δ^18^O are a mixture of δ^18^O_es_, and the unenriched δ^18^O of water in the transpiration stream, that is, δ^18^O_sw_ (Walker et al. 1989; Flanagan et al. 1994). To account for this, Farquhar and Lloyd (1993) incorporated a ‘Péclet effect’ into the CGD model by modelling δ^18^O of bulk leaf water (δ^18^O_lw_) as a function of transpiration rate:

(5) $${∆}^{18}O_{\mathrm{lw}}=\left(\delta^{18}O_{\mathrm{es}}-\delta^{18}O_{\mathrm{sw}}\right)\frac{1-e^{-℘}}{℘}$$

where the Péclet number (℘) is a function of transpiration rate (E), the effective pathlength of water molecule movement inside the leaf (L), the molar density of water (C) and the molecular diffusivity of H_2_^18^O in water (D):

(6) $$℘=\frac{\mathrm{EL}}{\mathrm{CD}}$$

and where E is given by:

(7) $$E=\frac{g_{s}\times \left(e_{i}-e_{a}\right)}{P}$$

where *P* is atmospheric pressure.

Thus, in the Péclet‐modified CGD model, δ^18^O_lw_ is controlled by the ratio of the advective transpiration flux of unenriched source water, and the back diffusion of ^18^O‐enriched water from the evaporating site. As the transpiration rate increases, the degree of back diffusion of ^18^O‐enriched water from the evaporating site mixing into the bulk leaf water decreases. Although studies indicate the presence of a Péclet effect in the leaf for many tree species, fewer species exhibit this δ^18^O gradient in the leaf than previously thought (Barbour et al. 2021).

The δ^18^O signal of sucrose in the leaf is equal to δ^18^O_lw_, plus an additional biochemical fractionation process (*ε*_bio_) of 27‰ that occurs during carbonyl hydration (Sternberg et al. 1986). Sucrose is then transported to the cambium where it is assimilated into cellulose. During cellulose synthesis, an estimated fraction of 0.4 (p_ex_) of the exchangeable oxygen in leaf sugars is exchanged with local xylem water at the site of cambial cellulose synthesis (Cernusak et al. 2005). Assuming that xylem water δ^18^O is close to δ^18^O_sw_, i.e., that the proportion (*p*_x_) of unenriched source water in local xylem water is 1, the *p*_ex_ *p*_x_ term is estimated as 0.4. (Roden et al. 2000; Cernusak et al. 2005; Barbour 2007). Resultantly, the isotopic signature of tree ring cellulose (δ^18^O_trc_) integrates both δ^18^O_sw_ and δ^18^O_lw_ signals (Barbour and Farquhar 2000):

(8) $$\delta^{18}O_{\mathrm{trc}}={∆}^{18}O_{\mathrm{lw}}\left(1-\left(p_{\mathrm{ex}}p_{x}\right)\right)+\varepsilon_{\mathrm{bio}}$$

**FIGURE 2:**
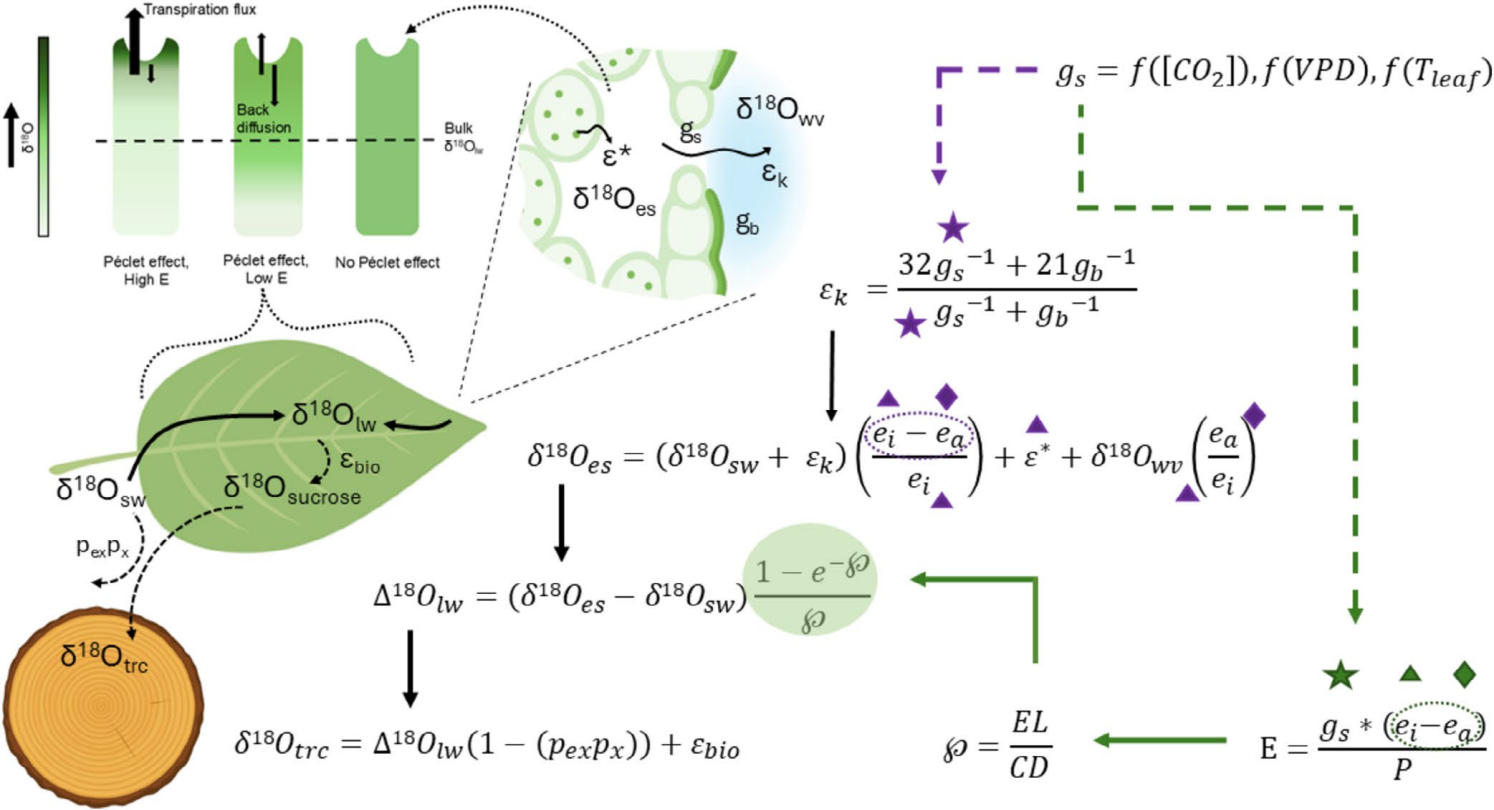
Models of leaf‐level processes and isotopic fractionations that contribute to δ^18^O_trc_. Depiction of how each isotope model (discussed in the main text) is linked to the oxygen isotope signal of tree ring cellulose. Icons (star, triangle, diamond, stippled circle) indicate the points at which g_s_, *T*_leaf_, e_a_ and VPD, respectively influence δ^18^O. Dashed arrows indicate the effects of g_s_. Purple arrow and icons indicate points at which the modelled variables affect δ^18^O with or without the Péclet effect. Green arrows and icons indicate points at which the modelled variables affect δ^18^O only when the Péclet effect is present. Diagrams illustrate the locations and processes in which variables in the equations affect δ^18^O. The top left visual demonstrates how transpiration rate (E) and the Péclet effect affect the degree of ^18^O‐enrichment at the evaporating site, and the degree to which this ^18^O‐enriched water pool contributes to bulk leaf water. Abbreviations are listed in Table 1.

These models are linked together as displayed in Figure 2.

**TABLE 1:** Abbreviations of all parameters used in the oxygen isotope models.

Abbreviation	Definition (unit)
δ^18^O	Deviation in the stable 18‐oxygen and 16‐oxygen isotope ratio between a sample and a standard reference (‰)
δ^18^O_sw_	δ^18^O of source water (‰)
δ^18^O_wv_	δ^18^O of atmospheric water vapour (‰)
δ^18^O_es_	δ^18^O of water at evaporating site of leaf (‰)
Δ^18^O_es_	δ^18^O enrichment of water at evaporating site above δ^18^O_sw_ (‰)
δ^18^O_lw_	δ^18^O of bulk leaf water (‰)
Δ^18^O_lw_	δ^18^O enrichment of bulk leaf water above δ^18^O_sw_ (‰)
δ^18^O_trc_	δ^18^O of tree ring cellulose (‰)
*ε*_*k*_	Kinetic fractionation of water during diffusion through the stomata and leaf boundary layer (‰)
ε*	Equilibrium fractionation between liquid water and vapour during evaporation (‰)
*ε*_bio_	Biochemical fractionation of cellulose synthesis (‰)
e_a_	Atmospheric vapour pressure (kPa)
e_*i*_	Leaf internal vapour pressure (kPa)
VPD	Leaf‐to‐air vapour pressure deficit = (e_i_ − e_a_) (kPa)
P	Atmospheric pressure (kPa)
g_s_	Stomatal conductance (mol m^−2^ s^−1^)
g_b_	Boundary layer conductance (mol m^−2^ s^−1^)
p_ex_	Proportion of oxygen in cellulose exchanged with medium water during cellulose synthesis
*p*_*x*_	Proportion of medium water at site of cellulose synthesis that is source water
E	Transpiration rate (mol m^−2^ s^−1^)
L	Effective pathlength (m)
C	Molar density of water (mol m^−3^)
D	Molecular diffusivity of water (m^2^ s^−1^)
*℘*	Péclet number

**FIGURE 3:**
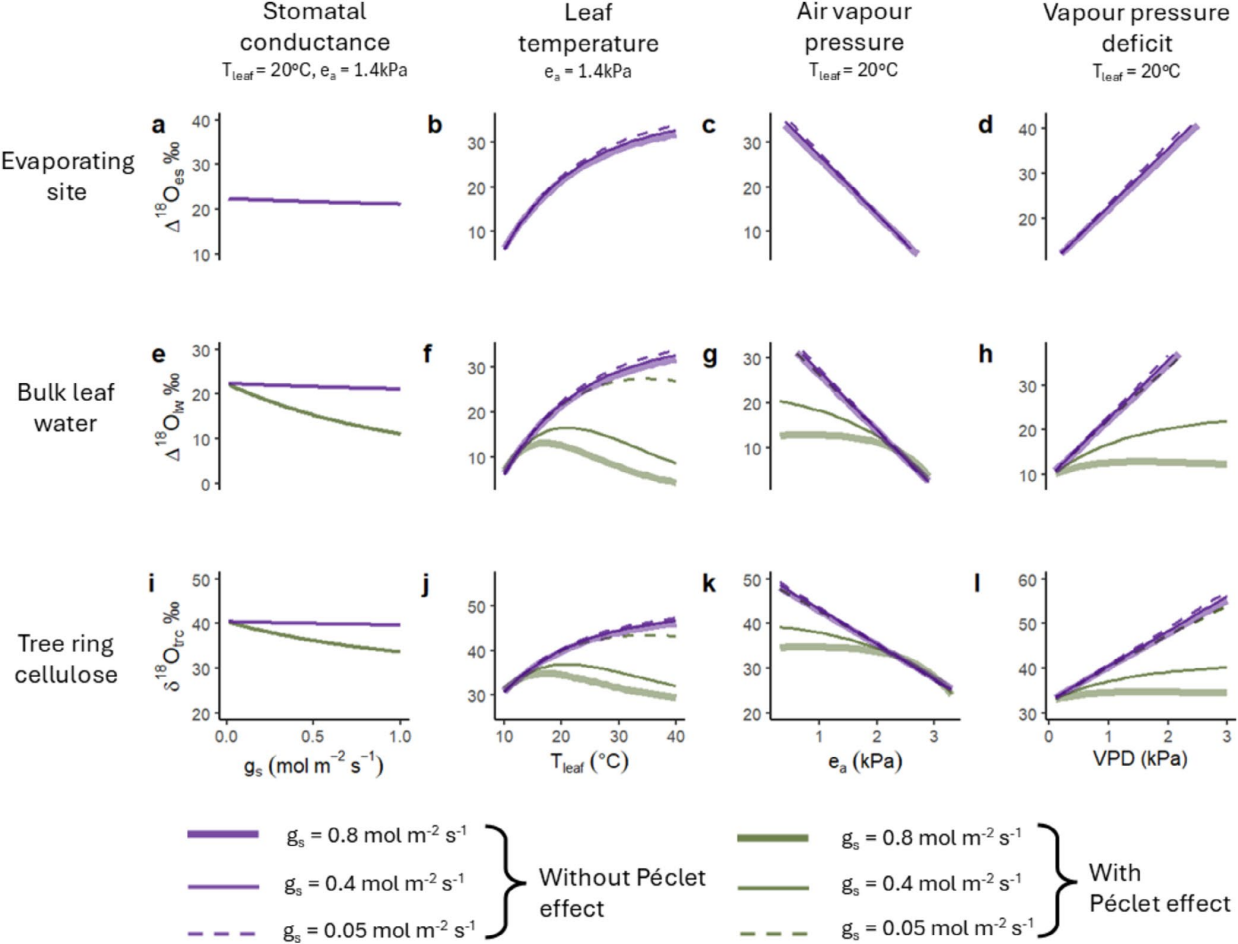
Model‐predicted effects of stomatal conductance (g_s_), leaf temperature (*T*_leaf_), atmospheric vapour pressure (e_a_) and leaf‐to‐air vapour pressure deficit (VPD) on δ^18^O in the leaf and cambium. The effects of each variable (each column) are simulated for δ^18^O above that of source water in the leaf evaporating site (top row), in bulk leaf water (middle row), and in tree ring cellulose (bottom row). Purple lines illustrate the effects of each variable in the CGD model without a Péclet effect, and green lines illustrate the effects of each variable in the Péclet‐modified CGD model. The different line types indicate the modelled effects for different g_s_ values. The model assumes that δ^18^O_sw_ and δ^18^O_wv_ are 0‰. Therefore, the enrichment above source water (Δ) is equal to the actual leaf water isotope values, i.e., Δ^18^O_es_ = δ^18^O_es_, and Δ^18^O_lw_ = δ^18^O_lw_. VPD is a more useful metric than e_a_ when considering the impact of transpiration on Δ^18^O_lw_. However, as VPD is a function of both T_leaf_ and e_a_, we include all three variables for clarity. The Y‐axis range for all plots is 30‰.

Figure 3 shows the theory‐predicted isolated effects of stomatal conductance (g_s_), leaf temperature (*T*_leaf_), atmospheric vapour pressure (e_a_) and leaf‐to‐air vapour pressure deficit (VPD) on δ^18^O at the evaporating site (Δ^18^O_es_, top row), on bulk leaf water (Δ^18^O_lw_, middle row), and on tree ring cellulose (δ^18^O_trc_, bottom row). We illustrate the effects of each variable between the extreme ends of plausible ranges for a typical temperate site, whilst varying g_s_ and T_leaf_ over credible ranges. We compare the effects of each variable both with and without the Péclet effect, and for three different g_s_ values, ranging from very low (0.05 mol m^−2^ s^−1^), to very high (0.8 mol m^−2^ s^−1^), for a temperate climate. The effects shown here do not include physiological plant responses to climate. For example, an increase in VPD would usually cause plants to partially close their stomata (i.e., g_s_ decreases) to limit water loss (Buckley 2019). However, for clarity we here only vary one parameter at a time, holding all other variables constant.

Theory indicates that stomatal conductance (g_s_) influences δ^18^O only via its effect on transpiration rate (E) caused by the Péclet effect (Figure 3e, purple vs. green line), and has an almost negligible effect on Δ^18^O_es_ (Figure 3a) via its impact on ε_k_ (Equation 3). In the presence of a Péclet effect, increasing g_s_ increases E which in turn increases the contribution of unenriched source water from the transpiration stream to bulk leaf water. This weakens the back‐diffusion of ^18^O‐enriched water from the evaporating site into bulk leaf water, thus lowering Δ^18^O_lw_ (Figure 3e).

Leaf temperature (*T*_leaf_) has a positive, non‐linear effect on Δ^18^O_es_ (Figure 3b). This is caused by two effects related to increases in *T*_leaf_, including small changes in ε* due to its temperature dependency and larger effects arising from increases in e_i_, which is assumed to be saturated. An increase in e_i_ increases the contribution of ε_k_ to Δ^18^O_es_. With the Péclet effect, the relationship between *T*_leaf_ and Δ^18^O_lw_ is bell‐shaped (Figure 3f). At lower temperatures, Δ^18^O_lw_ increases, due to increased enrichment at the site of evaporation, while at higher temperatures, Δ^18^O_lw_ decreases. This decrease at higher temperatures is driven by enhanced transpiration rates, resulting in a greater contribution of unenriched source water from the transpiration stream to the leaf. The magnitude of these effects varies depending on g_s_, as discussed at the end of this section.

The effect of atmospheric vapour pressure (e_a_) on Δ^18^O_es_ is negative and linear: as e_a_ increases, the contribution of ε_k_ to Δ^18^O_es_ decreases (Figure 3c). The Péclet effect dampens the effects of e_a_ at the evaporating site on Δ^18^O_lw_, due to increasing E, and thus an increasing contribution of unenriched source water from the transpiration stream to bulk leaf water. This effect varies depending on g_s_. In contrast, an increase of VPD has a positive linear effect on Δ^18^O_es_ (Figure 3d). The Péclet effect dampens the effects of VPD at the evaporating site on Δ^18^O_lw_ due to increasing E, and this effect also varies with g_s_.

In the presence of a Péclet effect, there is a significant interactive effect of g_s_ with *T*_leaf_, e_a_ and VPD. At larger g_s_, the effect of *T*_leaf_, e_a_ and VPD on Δ^18^O_lw_ weakens. When g_s_ is larger, E increases significantly more with increasing *T*_leaf_ and VPD (and decreasing e_a_), resulting in a larger flux of unenriched water into the leaf, which reduces the contribution of increasing Δ^18^O_es_ into bulk leaf water.

## 3 Methods

### 3.1 Sensitivity Analysis of Tree Ring δ^**18**^O to Changes in Stomatal Conductance and Climate

**FIGURE 4:**
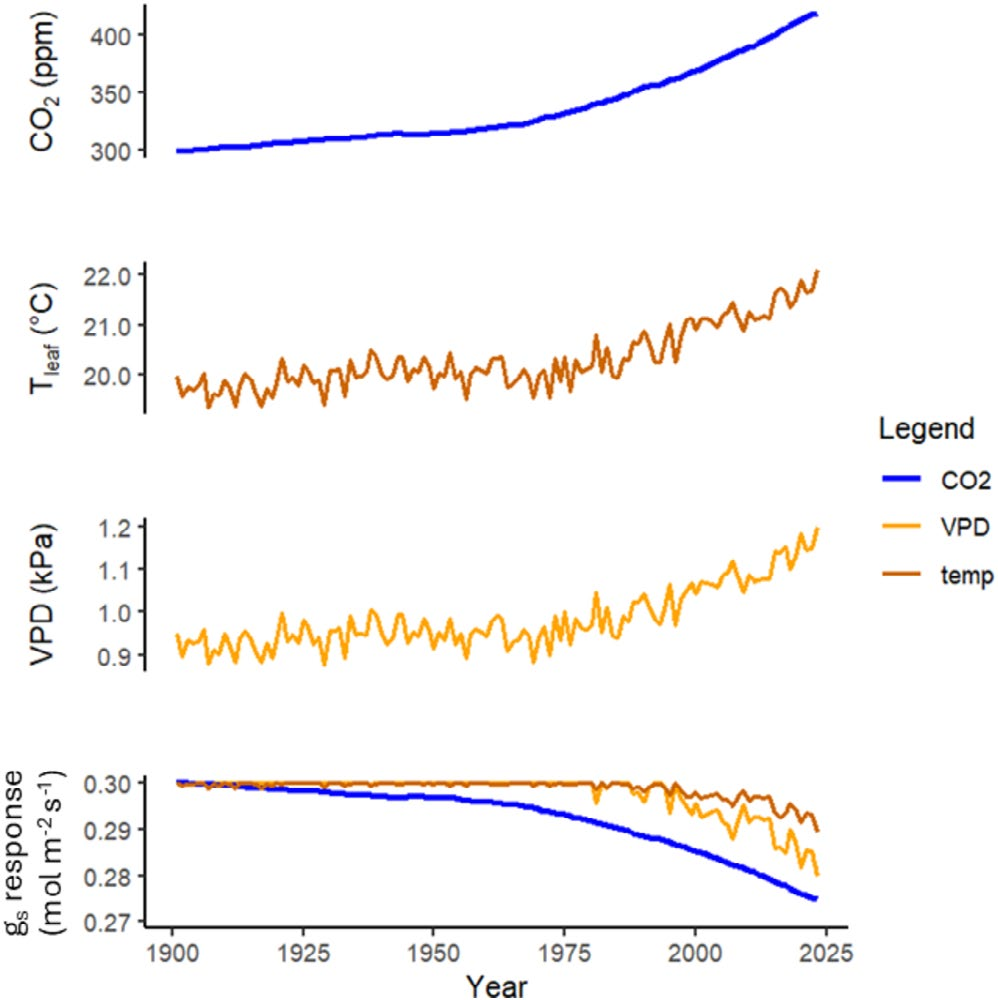
Historical climate and CO_2_ time series, and the modelled g_s_ responses used in the simulations. The simulated g_s_ changes (bottom panel) are shown for the first and second cases, with initial g_s_ of 0.3 mol m^−2^ s^−1^. The mean global annual atmospheric CO_2_ record (top panel) is obtained from a combined ice core dataset extended with data from the Mauna Loa Observatory (Keeling et al. 1976; Etheridge et al. 1996; Ballantyne et al. 2012). The modelled g_s_ response to CO_2_ (bottom panel—blue) is adapted from Walker et al. (2021) (Appendix S1: Eqn. SI.3.2). *T*_leaf_ (2nd panel) is modelled as 20°C plus the annual mean air temperature anomaly for all land points 23–67 N, from the GISTEMP 250 km surface temperature analysis record (GISTEMP Team 2025). Atmospheric Vapour Pressure (e_a_) is modelled as 1.4 kPa plus the annual mean e_a_ anomaly for all land points 23–67 N, from the CRU TS4.08 Vapour Pressure record (Harris et al. 2024). The VPD time series (3rd panel) is developed from the *T*_leaf_ and e_a_ models and is illustrated here for the first four cases. The modelled g_s_ responses to *T*_leaf_ (bottom panel—dark orange) and to VPD (bottom panel—light orange) are Stewart‐Jarvis functions (Jarvis 1976; Stewart 1988) (Appendix S1: Eqns. SI.3.3–4). The g_s_‐climate function used in model 2 (Table 2) combines the VPD and *T*_leaf_ response functions (i.e., g_s_ = *f* (VPD, *T*_leaf_)). The total g_s_ function used in models 3 and 4 (Table 2) combines all three response functions (i.e., g_s_ = *f* (CO_2_, VPD, *T*_leaf_)). Climate and CO_2_ data was downloaded using KNMI Climate Explorer (https://climexp.knmi.nl/).

The aim of this exercise is to assess the sensitivity of δ^18^O_trc_ trends to changes in g_s_ due to global changes since the beginning of the 20th century. We address this aim by modelling an average g_s_ response for an idealised mature tree growing in a Northern hemisphere temperate climate, first to changes in CO_2_ between 1901 and 2023 (i.e., g_s_ = *f* (CO_2_), c.f. Appendix S1: Eqn. SI.3.2), and then to changes in climate (i.e., g_s_ = *f* (*T*, VPD), c.f. Appendix S1: Eqns. SI.3.3–4) (Figure 4). We do not consider other climatic changes beyond changes in atmospheric vapour pressure (e_a_), temperature and VPD. Therefore, ‘climatic changes’ and ‘Δclimate’ refer only to changes in e_a_, temperature and VPD as occurred since 1901. Then, we simulate the δ^18^O response to the modelled changes in g_s_ (i.e., δ^18^O = f(g_s_), c.f. Figure 2), first at the leaf evaporating site (Δ^18^O_es_), then in bulk leaf water (Δ^18^O_lw_), and lastly in tree ring cellulose (δ^18^O_trc_). Note that we model ^18^O‐enrichment above source water (Δ^18^O), and that we assume δ^18^O_sw_ to be 0‰; thus, the real isotopic signal (δ^18^O) is the same value as expressions of the enrichment above source water. Models were developed and results analysed in RStudio version 4.5.1 (Posit Team 2025).

We examine the sensitivity of Δ^18^O_es_, Δ^18^O_lw_ and δ^18^O_trc_ trends to global changes between 1901 and 2023, by considering changes in g_s_ and changes in climate. To disentangle the isolated effects of CO_2_ and climate on g_s_ and to disentangle the indirect (i.e., g_s_‐moderated) effects on δ^18^O from the direct effects of climate, we developed four models (Table 2). Model 1 assesses the δ^18^O sensitivity to changes in g_s_ only due to increasing CO_2_. Thus, for model 1 we assume that changes in g_s_ are dependent only on CO_2_ concentration (Appendix S1: Eqn. SI.3.2), and changes in δ^18^O are only caused by this g_s_‐CO_2_ response. All other variables in the δ^18^O equations (Equations (1), (2), (3), (4), (5), (6), (7), (8)) are held constant at values given in Tables S1 and S2. Model 2 assesses the δ^18^O sensitivity to changes in g_s_ only due to climatic changes. Therefore, for model 2, we assume that changes in g_s_ are dependent only on temperature and VPD (i.e., g_s_ = *f* (*T*, VPD)), using Stewart‐Jarvis functions (Appendix S1: Eqns. SI.3.3–4). As VPD = (e_i_−e_a_), the effects of e_a_ on g_s_ are inbuilt into the g_s_‐VPD function. Thus, for model 2, changes in δ^18^O are only due to this g_s_‐climate response, and all other variables in the δ^18^O equations are held constant at values given in Tables S1 and S2. Model 3 assesses the δ^18^O sensitivity to the total anticipated changes in g_s_ due to both CO_2_ and climatic changes. Therefore, in model 3, we assume that changes in g_s_ are dependent on CO_2_, temperature and VPD (i.e., g_s_ = *f* (CO_2_, *T*, VPD)), and changes in δ^18^O are only due to the total g_s_ response. All other variables in the δ^18^O equations are held constant at values given in Tables S1 and S2. Lastly, model 4 assesses the δ^18^O sensitivity to the total anticipated changes in g_s_ due to both CO_2_ and climatic changes, plus the direct effects of climate on Δ^18^O_es_ in the CGD model (Equation 1) and on the rate of transpiration (Equation 7). Thus, model 4 assumes that changes in g_s_ are dependent on CO_2_, temperature and VPD, and that changes in δ^18^O are due to the total g_s_ response plus the changes in temperature, e_a_ and VPD directly in the δ^18^O equations. Other variables in the δ^18^O equations are held constant at values given in Table S2. Thus, model 4 effectively simulates the total expected change in tree ring δ^18^O.

**TABLE 2:** Model versions to elucidate the response of δ^18^O_trc_ to changes in g_s_ due to climate and CO_2_, and to the direct effects of climate.

Model		Description	Affected variables in isotope model
1	CO_2_ effects on g_s_	Changes in δ^18^O resulting from changes in g_s_ due to CO_2_ increases	All cases: *ε*_*k*_ via g_s_[CO_2_]
			Only cases with a Péclet effect: *℘* via g_s_[CO_2_]
2	Climate effects on g_s_	Changes in δ^18^O resulting from changes in g_s_ due to climatic changes	All cases: *ε*_*k*_ via g_s_[VPD, *T*]
			Only cases with a Péclet effect: *℘* via g_s_[VPD, *T*]
3	Total g_s_ effects	Changes in δ^18^O resulting from changes in g_s_ due to CO_2_ increases and climatic changes	All cases: *ε*_*k*_ via g_s_[CO_2_, VPD, *T*]
			Only cases with a Péclet effect: ℘ via g_s_[CO_2_, VPD, *T*]
4	Total g_s_ effects + direct climate effects	Changes in δ^18^O resulting from changes in g_s_ due to CO_2_ increases and climatic changes, plus direct effects of climatic changes in CGD model and on rate of transpiration	All cases: *ε*_*k*_ via g_s_[CO_2_, VPD, *T*] e_a_, e_*i*_[*T*], ε*[*T*]
			Only cases with a Péclet effect: ℘ via g_s_[CO_2_, VPD, *T*] and e_i_[*T*] + e_a_

We compare the δ^18^O response across six cases (three pairs of cases) for each of the four models (Appendix S1). For the first and second cases, we compare the sensitivity of δ^18^O for a species with, versus without, a Péclet effect, assuming an average g_s_ in 1901 (g_s0_) of 0.3 mol m^−2^ s^−1^. For the third and fourth cases, we compare the sensitivity of δ^18^O for a species with a high average g_s0_ (0.65 mol m^−2^ s^−1^), versus a species with a low average g_s0_ (0.05 mol m^−2^ s^−1^). These ‘high’ and ‘low’ g_s0_ values reflect a representative range observed for gymnosperm and angiosperm trees in temperate climates (Medlyn et al. 2001; Klein and Ramon 2019; Gardner et al. 2023). For the fifth and sixth cases, we compare the sensitivity of δ^18^O for a tree in a dry (40% RH and 1.41 kPa VPD in 1901), versus a wet climate (90% RH and 0.25 kPa VPD in 1901), with a g_s0_ of 0.3 mol m^−2^ s^−1^. All except the first case include a Péclet effect with a constant effective pathlength (L) of 20 mm.

## 4 Results

**FIGURE 5:**
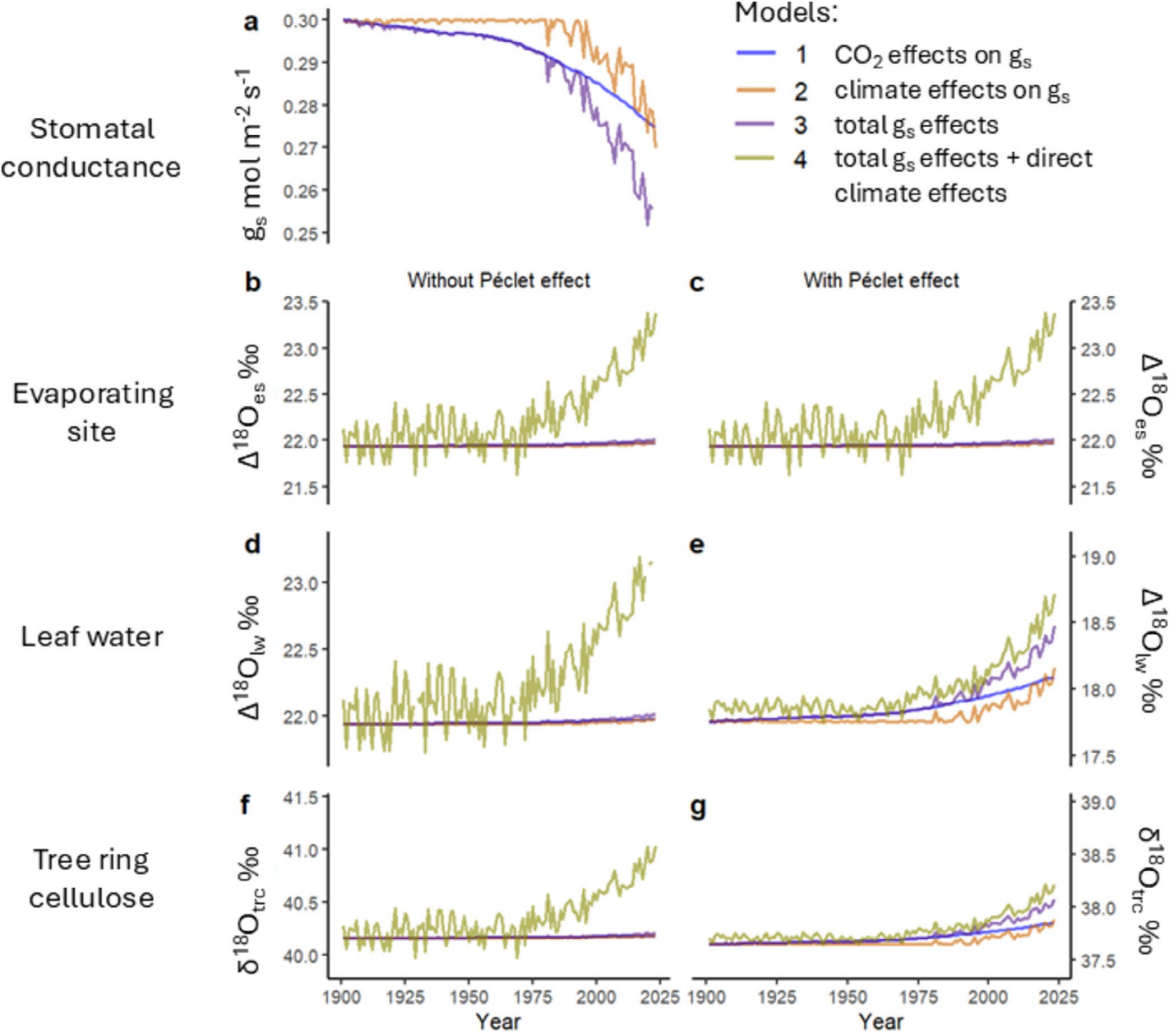
Predicted δ^18^O changes over the period 1901–2023, with and without the Péclet effect, to changes in g_s_ due to CO_2_ and climate, and to the direct effects of climate. Subplot a shows the modelled g_s_ response to CO_2_ (blue), climate (orange), and CO_2_ and climate (purple) for both cases. Subplots b‐g show the modelled δ^18^O response, without the Péclet effect (b, d, f), and with the Péclet effect (c, e, g), to changes in g_s_ and to the direct effects of climate under different levels of sensitivity (models 1–4) in Δ^18^O_es_ (b, c), in Δ^18^O_lw_ (d, e), and in δ^18^O_trc_ (f, g). Without the Péclet effect (left column), g_s_ only affects δ^18^O via *ε*_*k*_ (Equation 2), and VPD, e_a_ and temperature exert additional direct influences on δ^18^O via ^18^O‐enrichment at the evaporating site in model 4 only (Equation 1). With the Péclet effect (right column), g_s_ affects δ^18^O via *ε*_*k*_ (Equation 2) and *℘* (Equations 6 and 7), and, for model 4 only, VPD, e_a_ and temperature also influence δ^18^O via ^18^O‐enrichment at the evaporating site (Equation 1) and *℘* (Equations 6 and 7). The *y*‐axis range for subplots b, c is 2‰, and is 1.6‰ for subplots d–g.

We examined the contributions of changes in climate and CO_2_ between 1901 and 2023 to changes in stable oxygen isotope ratios at the site of evaporation (Δ^18^O_es_), in bulk leaf water (Δ^18^O_lw_), and in tree ring cellulose (δ^18^O_trc_). To disentangle the effects of plant physiology and climate on δ^18^O trends, we simulated the δ^18^O response to changes in g_s_ due to CO_2_ and climate, and to the direct effects of climate in the CGD model (Equation 1) and on the rate of transpiration (Equation 7). The climate variables in our model are e_a_, temperature and VPD, and therefore the effects of climate that we show here are limited to the changes in e_a_, temperature and VPD since 1901. Below, we first present time trends for the cases with and without the Péclet effect (Figure 5). We then provide a summary of these effects for three pairs of cases: with versus without Péclet effect (cases 1 vs. 2); low versus high g_s_ (cases 3 vs. 4); and dry versus wet climate (cases 5 vs. 6). Figures of the time trends for cases 3–6 are presented in Figures S4 and S5.

### 4.1 Effects of CO_2_ and Climate Change on Tree δ^18^O With and Without the Péclet Effect

According to the stomatal models (c.f. Appendix S1: Eqns. SI.3.2–4) based on compiled average g_s_‐CO_2_ responses (Walker et al. 2021) and Stewart‐Jarvis stomatal conductance functions (Jarvis 1976; Stewart 1988), increasing CO_2_, VPD and temperature cause g_s_ decreases between 0.025 and 0.053 mol m^−2^ s^−1^ (8%–18%) over the period 1901–2023, depending on the model used (Figure 5a). These model‐predicted decreases in g_s_ result in an increase in δ^18^O_trc_ between 0.02‰ and 0.43‰ (Figure 5f,g, models 1–3). When the direct effects of climate are also taken into account, δ^18^O_trc_ increases between 0.51‰ and 0.75‰ (model 4).

Without the Péclet effect, changes in g_s_ only influence the kinetic fractionation factor (ε_k_), which has a very small effect in the CGD model (Equation 1). Resultantly, decreases in g_s_ lead to negligible effects on Δ^18^O_es_ (Figure 5b,d,f, models 1–3). In contrast, when the Péclet effect is present, decreases in g_s_ lead to small increases in leaf water ^18^O‐enrichment (Figure 5e, models 1–3). This effect arises due to a reduced transpiration flux, allowing greater back‐diffusion of ^18^O‐enriched water from the evaporating site into bulk leaf water.

The largest effect on δ^18^O is the direct effect of climate, independent of changes in g_s_. Leaf warming and decreases in e_a_ elevate VPD, which also enhances the contribution of ε_k_ to the isotopic signal at the site of evaporation, leading to an increase of Δ^18^O_es_ by 1.18‰ (Figure 5, model 4). The direct climate effects most strongly affect Δ^18^O_lw_ and δ^18^O_trc_ in the case without a Péclet effect (Figure 5d,f, model 4). In contrast, enrichment at the site of evaporation is weakened in Δ^18^O_lw_ and δ^18^O_trc_ in the case with a Péclet effect (Figure 5c,e,g, model 4). This is because increases in VPD increase the flux of unenriched water from the transpiration stream into the leaf, which weakens the back‐diffusion of ^18^O‐enriched water from the evaporative site.

### 4.2 Effects of CO_2_ and Climate Change on Tree δ^18^O Across Cases

#### 4.2.1 Stomatal Responses to CO_2_ and Climate Change

**FIGURE 6:**
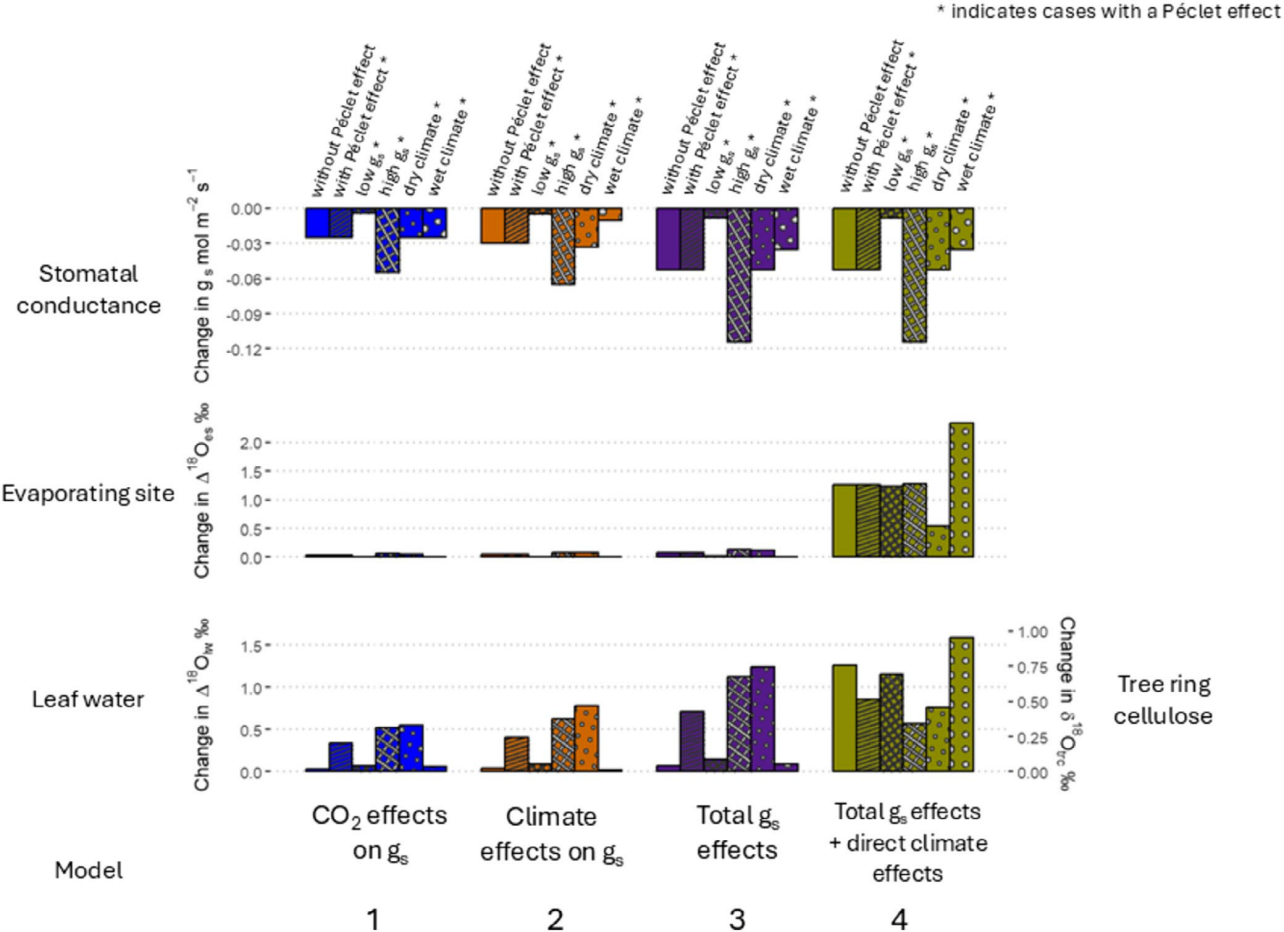
Modelled net changes between 1901 and 2023 in stomatal conductance and δ^18^O for each of the six cases, across the four models. Changes are shown for six cases, indicated at the top of the panel (without Péclet effect—blank, with Péclet effect—stripe, low g_s_—thin crosshatch, high g_s_—thick crosshatch, dry climate—small dot, wet climate—large dot). For all panels, each bar illustrates the predicted *net change* between 1901 and 2023 in g_s_ (top), Δ^18^O_es_ (middle), Δ^18^O_lw_ (bottom—left) and δ^18^O_trc_ (bottom—right) for each case due to: CO_2_ effects on g_s_ (model 1—blue), climate effects on g_s_ (model 2—orange), total, i.e., CO_2_ and climate, effects on g_s_ (model 3—purple), and total effects on g_s_ plus the direct effects of climate in the CGD model (Equation 1) and on the rate of transpiration (Equation 7) (model 4—yellow).

Predicted decreases in g_s_ from 1901 to 2023 vary between cases and models (Figure 6, top panel). In model 1, where we only vary g_s_ in response to CO_2_ increases from 1901 to 2023, g_s_ is reduced by 8%. The absolute changes are smaller (−0.004 mol m^−2^ s^−1^) for the low g_s_ case, and larger (−0.055 mol m^−2^ s^−1^) for the high g_s_ case.

Increases in temperature and VPD from 1901 to 2023 have an effect on g_s_ that is of comparable magnitude as the effect of CO_2_ (Figure 6, top row, models 2 vs. 1). VPD is larger in the dry climate case, resulting in a greater decrease in g_s_ (−0.033 mol m^−2^ s^−1^), compared to the wet climate case (−0.011 mol m^−2^ s^−1^).

For each case, models 3 and 4 have the same simulated stomatal responses that reflect the combined effects of CO_2_ and climatic changes on g_s_ (Figure 6, top panel, models 3,4). From 1901 to 2023, the total modelled decrease in g_s_ is 12% for the wet climate case, and 18% for all other cases. This results in the largest change in g_s_ of −0.114 mol m^−2^ s^−1^ for the high g_s_ case, and the smallest change in g_s_ of −0.009 mol m^−2^ s^−1^ for the low g_s_ case.

#### 4.2.2 Predicted Changes of δ^18^O at the Leaf Evaporating Site, in Bulk Leaf Water and in Tree Ring Cellulose, in Response to Global Change

For all cases, changes in g_s_ between 1901 and 2023 have a negligible effect on Δ^18^O_es_ (Figure 6, middle panel, models 1–3). In contrast, the direct effects of climate, independent from g_s_, cause relatively large increases in Δ^18^O_es_ between 0.43‰ and 2.32‰ (model 4).

In bulk leaf water and at the tree ring level, increasing CO_2_ and/or changes in climate from 1901 to 2023 only cause increases in δ^18^O when the Péclet effect is present (Figure 6, bottom panel, models 1–3). Furthermore, these effects on δ^18^O are very weak for trees with low average g_s_ and in wet climates, again due to the Péclet effect.

The increase in Δ^18^O_lw_ is significantly larger for the high g_s_ case compared to the low g_s_ case, because absolute changes in g_s_ due to CO_2_ and climate are larger for trees with high g_s_ (Figure 6, top and bottom panels, models 1–3). The larger decrease in g_s_ for the high g_s_ case results in a greater decrease in leaf transpiration rate. Resultantly, there is a greater increase in the back‐diffusion of ^18^O‐enriched water from the evaporating site into the bulk leaf water in the high g_s_ case compared to the low g_s_ case.

VPD is larger in the dry climate case compared to the wet climate case; therefore, the decrease in g_s_ between 1901 and 2023 results in a greater decrease in leaf transpiration rate (Equation 7). Thus, there is a greater increase in the back‐diffusion of ^18^O‐enriched water from the evaporating site into the bulk leaf water for the dry climate case, resulting in a larger increase in Δ^18^O_lw_ compared to the wet climate case (Figure 6, bottom panel, model 1).

#### 4.2.3 The Direct Effects of Climate Change on Tree δ^18^O

Despite the same modelled changes in g_s_, absolute changes in δ^18^O_trc_ for each case differ significantly between models 3 and 4 (Figure 6, bottom panel, columns 3 vs. 4). For the ‘without Péclet effect’, ‘low g_s_’ and ‘wet climate’ cases, there are large increases in the observed δ^18^O_trc_ signals (model 4), compared to the predicted increases in δ^18^O_trc_ due to changes in g_s_ alone (model 3). This is because there are large increases in Δ^18^O_es_ due to the effects of warming and increased VPD between 1901 and 2023, according to the CGD model (Equation 1).

For the high g_s_ and dry climate cases, there are smaller increases in δ^18^O_trc_ for model 4 compared to model 3 (Figure 6, bottom panel, columns 3 vs. 4). This is due to the counteracting effects of decreasing g_s_ and increasing VPD in Equation (7) for model 4, which weakens the decrease in leaf transpiration rate compared to model 3. Resultantly, for model 4, there is a smaller increase in the back‐diffusion of ^18^O‐enriched water from the evaporating site into bulk leaf water.

#### 4.2.4 Sensitivity of Tree Ring δ^18^O to Changes in Stomatal Conductance

Across cases, the sensitivity of δ^18^O_trc_ to a given decrease in g_s_ varies (Table 3). For the case without a Péclet effect, δ^18^O_trc_ increases by 0.86‰ per 1 mol m^−2^ s^−1^ decrease of g_s_, i.e., Δδ^18^O_trc_/Δg_s‐total_ = −0.86‰ (mol m^−2^ s^−1^)^−1^. For the case with a Péclet effect, Δδ^18^O_trc_/Δg_s‐total_ = −8.14‰ (mol m^−2^ s^−1^)^−1^. With all other conditions being the same for these two cases, the sensitivity of δ^18^O_trc_ to a given change in g_s_ is 9.5× greater when there is a Péclet effect in the leaf (Table 3, columns 1 vs. 2). The sensitivity of δ^18^O_trc_ to a given change in g_s_ is 1.8× greater for the low g_s_ case (g_s0_ = 0.05 mol m^−2^ s^−1^) compared to the high g_s_ case (g_s0_ = 0.65 mol m^−2^ s^−1^) (Table 3, columns 3 vs. 4). Nonetheless, according to our model, changes in g_s_ due to CO_2_ and climate are larger for the high g_s_ case, such that changes in δ^18^O_trc_ are also greater compared to the low g_s_ case (Figure 6, bottom panel, models 1–3). For the same change in g_s_, the sensitivity of δ^18^O_trc_ is 9.3× greater for the dry climate case (40% RH in 1901) compared to the wet climate case (90% RH in 1901) (Table 3, columns 5 vs. 6).

**TABLE 3:** Sensitivity of δ^18^O_trc_ to modelled changes in g_s_ for each case.

	Change in δ^18^O_trc_ per change in g_s_ (Δδ^18^O_trc_/Δg_s_) ‰ (mol m^−2^ s^−1^)^−1^					
	Standard	Standard	Low g_s_	High g_s_	Wet climate	Dry climate
	Without Péclet effect	With Péclet effect				
Sensitivity to CO_2_ effects on g_s_ Δδ^18^O_trc_/Δg_s‐CO2_	−0.85	−8.01	−10.38	−5.72	−1.53	−13.15
Sensitivity to climate effects on g_s_ Δδ^18^O_trc_/Δg_s‐climate_	−0.85	−8.04	−10.38	−5.75	−1.52	−14.07
Sensitivity to total g_s_ effects Δδ^18^O_trc_/Δg_s‐total_	−0.86	−8.14	−10.38	−5.89	−1.53	−14.28

## 5 Discussion

### 5.1 Results Summary

We find that changes in g_s_ due to CO_2_ and climate between 1901 and 2023 only significantly contribute to δ^18^O_trc_ trends for species with a pronounced Péclet effect. Indeed, we find that δ^18^O_trc_ is 9.5× more sensitive to changes in g_s_ when the Péclet effect is present. This is because changes in g_s_ only have a significant effect on leaf water ^18^O‐enrichment by causing changes in transpiration flux (Equation 7) which moderates the degree of back‐diffusion of ^18^O‐enriched water from the evaporating site into bulk leaf water (Equation 6). Although δ^18^O_trc_ is 1.8× more sensitive to a given change in g_s_ for species with a low average g_s_, changes in g_s_ are easier to detect in δ^18^O_trc_ trends for species with a high average g_s_ because CO_2_ and climate cause larger changes to g_s_. These species with larger g_s_ changes exhibit larger changes in δ^18^O_trc_ because of the greater reduction in transpiration flux, resulting in greater increases in bulk leaf water δ^18^O. This is of course only true under the assumption that g_s_ responses are scaled proportionately to average g_s_. Lastly, we find that δ^18^O_trc_ is 9.3× more sensitive to the same changes in g_s_ for a tree situated in a dry climate versus a wet climate. This is because VPD is greater in drier climates, and thus, a given change in g_s_ causes a greater change in transpiration rate in a dry compared to a wet (i.e., low VPD) climate (Equation 7). Resultantly, in drier climates, a given change in g_s_ corresponds to a greater change in the degree of back‐diffusion of ^18^O‐enriched water from the evaporating site into the bulk leaf water (Equation 6).

### 5.2 Key Issues for Using Tree Ring δ^18^O to Infer Long‐Term Stomatal Conductance Trends

Our results show that there are three main issues associated with detecting climate and CO_2_‐induced changes in g_s_ from δ^18^O_trc_ trends. These issues are in addition to the errors associated with estimates of historical source water δ^18^O trends, as addressed by Lin et al. (2022). First, changes in δ^18^O_trc_ caused by changes in g_s_ are small compared to the 0.3‰ measurement precision (Boettger et al. 2007). The second and most significant issue is that changes in δ^18^O_trc_ caused by changes in g_s_ are small in comparison to changes in δ^18^O_trc_ caused by the direct effects of increasing VPD and temperature in the CGD model (Equation 1) and the Péclet effect (Equation 6). A third related issue is the high interannual variability of VPD and temperature superimposed on long‐term increases in these variables, which adds further complexity to disentangling a g_s_ signal from δ^18^O_trc_ trends. Thus, without independent, reliable estimates of VPD and temperature, detecting long‐term changes in g_s_ from δ^18^O_trc_ trends is not possible.

### 5.3 Implications of Our Findings

Our results have significant implications for the conclusions in Mathias and Thomas (2021) and Guerrieri et al. (2019). The studies use tree ring δ^13^C and δ^18^O_trc_ trends to conclude that increases in iWUE under anthropogenic climate change and rising CO_2_ levels are primarily due to increases in assimilation rate (A), whereas g_s_ remains effectively unchanged or only decreases in water‐limited sites. However, the results of our simulation cases suggest that changes in g_s_ due to CO_2_ and climate are only detectable in δ^18^O_trc_ trends for trees growing in dry climates, and not in wet climates. Thus, it is well possible that g_s_ has decreased in more sites than recognised in these studies but was simply not detectable in the observed δ^18^O_trc_ trends. This reasoning is previously demonstrated in sensitivity analyses whereby plant δ^18^O is modelled as a function of g_s_ and RH, demonstrating that δ^18^O is significantly more responsive to the same change in g_s_ under low RH, i.e., dry, moisture‐limited conditions, versus under high RH, i.e., wet conditions (Roden and Siegwolf 2012; Barbour et al. 2000). Experimental evidence indeed demonstrates that changes in g_s_ are more detectable in plant δ^18^O in drier conditions. For example, δ^18^O of cellulose in cotton leaves was more sensitive to decreases in g_s_ under 43% RH versus under 76% RH (Barbour and Farquhar 2000). However, it is important to note that the conclusions that g_s_ did not change for most sites in Mathias and Thomas (2021) and Guerrieri et al. (2019) may still be correct. Indeed, it is reasonable to expect based on physiological principles that trees would avoid reducing g_s_ when water is not a limiting factor, in order to maintain their supply of CO_2_ into the leaf for photosynthetic assimilation. However, our results demonstrate that δ^18^O_trc_ trends cannot be used to detect the small decreases in g_s_ as expected from increases in CO_2_ since pre‐industrial levels. Given these current methodological limitations, we strongly caution against such applications of δ^18^O_trc_ trends to infer long‐term changes in g_s_ as a result of changes in CO_2_.

Our study further exemplifies that the effects of increasing VPD and temperature at the leaf evaporating site have a significantly greater impact on δ^18^O_trc_ and Δ^18^O_lw_ trends than do the effects of changes in g_s_ due to CO_2_ and climate. Although it would be plausible to infer decreases in g_s_ from increases in δ^18^O_trc_ caused primarily by long‐term trends of increasing VPD, this is not a direct detection of changes in g_s_. Indeed, it is also plausible that g_s_ may not change under long‐term VPD increases. For example, g_s_ may acclimatise to long‐term exposures of rising VPD (Marchin et al. 2016). Additionally, more anisohydric tree species, that can withstand highly negative water potentials and are thus less vulnerable to xylem embolism, may opt to keep their stomata open under increasing VPD and diminishing plant water status to maintain carbon gain and growth (Tardieu and Simonneau 1998; McDowell et al. 2008). Thus, a clear VPD‐driven δ^18^O_trc_ trend is not a conclusive indicator of a long‐term g_s_ trend because VPD can affect δ^18^O_trc_ without affecting g_s_ (c.f., Figure 6, models 3 vs. 4). Disentangling the effects of g_s_ changes on δ^18^O_trc_ trends is further complicated because the high interannual variability of VPD and temperature causes high interannual variability of δ^18^O_trc_ signals (see Figure 5). Therefore, long‐term increases and year‐to‐year variation in VPD and atmospheric temperatures present significant challenges for attempts to correctly attribute the changes in δ^18^O_trc_ that are due to changes in g_s_.

### 5.4 Realism and Limitations of the Model

Our analysis shows that the existing methods to use δ^18^O_trc_ trends to predict how g_s_ is changing with rising CO_2_ levels and climatic changes are too uncertain to currently be used (Lin et al. 2022; Roden et al. 2022). This finding is relevant for interpreting tree ring δ^18^O trends because our simulations compare well with observed trends in published δ^18^O_trc_ records (Appendix S6), indicating that the parameters of the model and simulations are within the realm of observations from field studies. Despite the relatively large variation in δ^18^O_trc_ changes between studies, potentially caused in part by variation in δ^18^O_sw_ changes, the 50‐year change in δ^18^O_trc_ predicted by our model is close to the mean δ^18^O_trc_ change for 172 global tree ring chronologies from 136 sites and 15 tree species (Guerrieri et al. 2019; Mathias and Thomas 2021; Treydte et al. 2023) (Figure S6). Thus, the issues associated with using δ^18^O_trc_ trends to infer long‐term changes in g_s_ as revealed by our study reflect real issues that can lead to erroneous interpretations of δ^18^O_trc_ records.

There are some limitations to the modelling assumptions that we use for this analysis although they are not critical to our overall conclusions. Firstly, the focus of this analysis is on temperate climates, which may limit the applicability of our findings. Therefore, we checked whether our findings also hold for warmer, tropical climates (Appendix S7). We find similar trends and magnitudes of change in δ^18^O_trc_ across cases and models for the tropical and temperate simulations (Figure S7i,ii), indicating that it is also not possible to unambiguously disentangle long‐term g_s_ trends from tropical δ^18^O_trc_ records.

Secondly, Stewart‐Jarvis functions were used to approximate g_s_ responses to increasing temperatures and VPD between 1901 and 2023. However, g_s_ is sensitive to other variables such as light and soil water potential which were part of the original Stewart‐Jarvis model (Jarvis 1976; Stewart 1988). We did not use these functions in our simulations, first because we do not expect light intensity to have changed significantly over the last 100 years, and second because simulating soil water trends requires site‐specific assumptions of the soil properties and the implementation of complex hydraulic models which are beyond the capacity and purpose of this study. Finally, Stewart‐Jarvis functions are constructed based on diurnal responses of g_s_ to environmental variables, while we use these functions to model long‐term g_s_ responses. Thus, if g_s_ does acclimatise to long‐term increases in temperature and VPD (Marchin et al. 2016), then g_s_ changes are expected to be smaller than modelled, reinforcing our core message that g_s_ responses to anthropogenic global changes are too small to significantly contribute to observed δ^18^O_trc_ trends. In contrast, the direct effects of climate have a far greater contribution to changes in δ^18^O_trc_. Therefore, any small δ^18^O_trc_ trend due to long‐term g_s_ changes is obscured by larger δ^18^O_trc_ trends driven by the direct effects of increasing VPD and temperature in Equations (1) and (7), and also by noisiness in the δ^18^O_trc_ signal due to interannual climatic variability.

The modelled g_s_ response to CO_2_ is derived from the C3 plant CO_2_‐g_s_ response curve in Walker et al. (2021), which integrates observed CO_2_‐g_s_ relationships from a wide range of studies, and which sits within the range of stomatal sensitivities to CO_2_ measured in laboratory settings for 57 angiosperm and gymnosperm tree species (Klein and Ramon 2019). Thus, our model should reflect a general g_s_‐CO_2_ response in trees, although this will vary between species and with other tree physiological parameters (e.g., tree size, age and health). For example, some studies find weaker, or even negligible, g_s_ responses to changes of CO_2_ in mature trees of temperate regions (Keel et al. 2007; Bader et al. 2013; Streit et al. 2014; Klein et al. 2016).

### 5.5 Further Uncertainties, Recommendations for Future Studies, and Methodological Improvements

There are additional considerations that we did not address in this study, but which cause further uncertainties for the use of δ^18^O_trc_ trends as a proxy for long‐term g_s_ changes. The effective pathlength (L) in the Péclet term varies between species (Kahmen et al. 2009). Moreover, there is evidence that L can vary within a species with changes in transpiration rate and needle age (Song et al. 2013; Roden et al. 2015). If this is the case, the response of Δ^18^O_lw_ and δ^18^O_trc_ to changes in g_s_ is further dampened, as demonstrated in Lin et al. (2022). Additionally, there is evidence that several environmental and physiological factors may drive variation in the proportion of oxygen that exchanges with source water during cambial cellulose synthesis (*p*_ex_ *p*_x_). This degree of exchange has been shown to be dependent on the type of species (Wang et al. 1998; Gessler et al. 2013), the time and transport distance between sucrose synthesis in the leaf and its incorporation into cambial cellulose (Farquhar et al. 1998; Barbour and Farquhar 2000; Barnard et al. 2007; Song et al. 2014), the site aridity (Cheesman and Cernusak 2016) and CO_2_ concentration (Morgner et al. 2024). The δ^18^O_trc_ model (Equation 8) assumes that a constant fraction (0.4) of the oxygen in cellulose is exchanged with source water based on experimental evidence (Roden and Ehleringer 1999; Cernusak et al. 2005). Resultantly, unaccounted variability in p_ex_p_x_ may result in erroneous interpretations of Δ^18^O_lw_ (and thus, g_s_) from δ^18^O_trc_ records.

Future research efforts should investigate the impact of these remaining uncertainties on interpretations of g_s_ from long‐term in situ δ^18^O_trc_ trends, e.g., by repeating a similar sensitivity analysis that simulates δ^18^O_trc_ trends with L and *p*_ex_ *p*_x_ varying with climatic changes and transpiration rate. Experimental studies would be particularly useful to assess whether the proportion of oxygen exchanged with source water during cambial cellulose synthesis (i.e., *p*_ex_ *p*_x_) indeed changes with long‐term exposures to CO_2_ and climatic changes. Additionally, the research field would benefit from a large‐scale quantification of Péclet effects across tree species, sites and climatic conditions, and from identifying how well the Péclet model predicts leaf δ^18^O gradients averaged over growing seasons. Lastly, we suggest experimentally validating the results of this study, for example by comparing g_s_ and Δ^18^O_lw_ measurements between ambient and elevated CO_2_ conditions at Free Air CO_2_ Enrichment (FACE) facilities (e.g., Battipaglia et al. 2013). Such experiments could provide insight into whether CO_2_‐induced changes in g_s_ are indeed indetectable in Δ^18^O_lw_, and thus, in δ^18^O_trc_. These, and further research recommendations are summarised in Table 4.

**TABLE 4:** Unresolved issues for δ^18^O_trc_‐derived estimates of g_s_ changes and suggested future experiments.

Issue	Explanation	Recommendations for future research
Long‐term temporal source water δ^18^O trends	Lack of long‐term records of changes in δ^18^O_sw_ used by trees	Explore whether position‐specific δ^18^O_trc_ approaches (e.g., Sternberg et al. 2003, 2007; Waterhouse et al. 2013) allow to calculate δ^18^O_sw_ incorporated into annual tree ring cellulose Collect and analyse long‐term rainfall δ^18^O (δ^18^O_p_) at more sites (c.f. GNIP), to understand how δ^18^O_p_ varies with climate, which may allow improving historical reconstructions
Péclet effect and effective pathlength (L)	Unknown degree of Péclet effects for many tree species Unknown effects of L variability on bulk leaf δ^18^O	Quantification of Péclet effects across more tree species In situ observations of how leaf δ^18^O varies temporally (diurnally, seasonally) and between species
Proportion of oxygen in cellulose exchanged with source water during cellulose synthesis (*p*_ex_ *p*_x_)	Unclear how p_ex_p_x_ varies across and within species under environmental changes	In situ isotope labelling and tracing studies to quantify degree of oxygen exchanged with source water over the growing season and in response to climatic changes
Empirical evidence of how CO_2_ and climate‐induced g_s_ changes contribute to δ^18^O_trc_ trends	Lack of empirical evidence of CO_2_ and climate‐induced stomatal changes having driven changes in δ^18^O_lw_ and δ^18^O_trc_ in field observations	Utilisation of CO_2_ fertilisation (FACE) experiments to analyse g_s_ and Δ^18^O_lw_ trends between ambient and elevated CO_2_ levels under in situ conditions

Abbreviation: GNIP, Global Network of Isotopes in Precipitation (www.iaea.org/services/networks/gnip).

Despite the significant limitations for current methods attempting to derive changes in g_s_ from δ^18^O_trc_ trends, steps may be taken to improve the detection of changes in g_s_ using this approach. Firstly, our results show that δ^18^O_trc_ is most sensitive to a given change in g_s_ for species with a Péclet effect and a high average g_s_, and for trees in dry climates. Thus, assuming the issues regarding the effects of climatic changes on δ^18^O_trc_ trends can also be addressed, preliminary considerations can be made to select tree species and sites where δ^18^O_trc_ would be most sensitive to potential g_s_ changes. For example, leaf hydraulic design (Zweiniecki et al. 2007) may moderate the presence of a Péclet effect in the leaf (Holloway‐Phillips et al. 2016; Barbour et al. 2021, 2024). Of the species tested in Barbour et al. (2021, 2024), the hydraulic designs of gymnosperms are generally not associated with whole‐leaf Péclet effects, whereas the hydraulic designs of angiosperms are generally conducive to whole‐leaf Péclet effects. Although these implications need to be tested in situ and for more species, these findings alongside the results of our study suggest that long‐term g_s_ trends may be better detected in δ^18^O_trc_ chronologies of angiosperms with hydraulic designs that facilitate whole‐leaf Péclet effects. Site‐level considerations to optimise the detection of long‐term changes in g_s_ from δ^18^O_trc_ trends should include selecting drier regions with minimal year‐to‐year climatic variability, and avoiding sites predisposed to environmental stressors such as low nutrient availability, long‐term drought and pollution (see Siegwolf et al. 2023 for a comprehensive overview of environmental considerations).

Another potentially promising avenue to improve the detection of g_s_ changes from δ^18^O_trc_ trends is position‐specific δ^18^O analysis (i.e., analysis of δ^18^O at each oxygen position in the cellulose repeating unit). This method could allow disentangling δ^18^O_sw_ and δ^18^O_lw_ signals from tree ring cellulose, and would thus be able to overcome the interfering influence of variation in source water δ^18^O over time. There is evidence that specific positions of the cellulose monomeric unit undergo complete exchange with xylem water during heterotrophic cellulose synthesis in wheat, whereas other positions do not exchange with xylem water (Waterhouse et al. 2013; Sternberg et al. 2003). Thus, positions where there is complete exchange reflect a pure δ^18^O_sw_ signal, and positions where there is no exchange retain δ^18^O of the material from which the cellulose was formed. It remains yet to be tested whether exchange with xylem water also occurs at specific positions of the monomeric unit during cambial cellulose synthesis. If similar exchange processes also occur for trees, then some positions of the tree ring cellulose monomeric unit could reflect a true δ^18^O_sw_ signal, and other positions could reflect a true δ^18^O_lw_ signal. A position‐specific approach suitable for tree ring studies could improve the ability to detect longer trends of changes in g_s_ in two important ways. Firstly, Δ^18^O_lw_ is nearly two times more sensitive than δ^18^O_trc_ to changes in g_s_ (Figure 6). Secondly, this approach would provide actual δ^18^O_sw_ values used by the plant. Therefore, this would avoid the use of untested and unreliable isotope‐climate models to reconstruct historical trends in δ^18^O_sw_, which are required to calculate Δ^18^O_lw_ and g_s_.

## 6 Conclusions

Using current tree ring oxygen isotope models, we find that using long‐term δ^18^O_trc_ trends is not a suitable method to infer g_s_ responses to CO_2_ and climate change, because in many cases the sensitivity of δ^18^O_trc_ trends to changes in g_s_ is too weak to be detectable. Without a Péclet effect, δ^18^O_trc_ is unresponsive to changes in g_s_, and even when a Péclet effect is included, δ^18^O_trc_ is significantly less sensitive to changes in g_s_ in wetter climates, compared to the same g_s_ change in a dry climate. Thus, even when there is no significant trend in δ^18^O_trc_, g_s_ may have changed but simply did not elicit a large enough change in δ^18^O_trc_ to be detected. Yet, even in contexts where δ^18^O_trc_ is sufficiently responsive to changes in g_s_, the enrichment effects of VPD and temperature at the leaf evaporating site have a far greater effect on δ^18^O_trc_, such that any trends in δ^18^O_trc_ cannot be unambiguously attributed to CO_2_ or climate‐driven changes in g_s_. Thus, we do not recommend the use of δ^18^O_trc_ trends as a suitable indicator for long‐term responses of g_s_ to CO_2_ and climatic changes.

## Author Contributions

**Imogen Carter:** conceptualization, data curation, formal analysis, methodology, writing – original draft, writing – review and editing. **Roel Brienen:** conceptualization, supervision, writing – review and editing. **Manuel Gloor:** conceptualization, supervision, writing – review and editing.

## Ethics Statement

The authors have nothing to report.

## Consent

The authors have nothing to report.

## Conflicts of Interest

The authors declare no conflicts of interest.

## Supporting information


**Data S1:** gcb70604‐sup‐0001‐DataS1.pdf.

## Data Availability

The data and code that support the findings of this study are openly available in Dryad at https://doi.org/10.5061/dryad.905qftv01.
