# Supplementary material for: Can Oxygen Isotopes in Tree Rings Be Used to Detect Stomatal Responses to Global Change?
Source: Glob Chang Biol. 2025 Nov 15;31(11):e70604. doi: 10.1111/gcb.70604 (PMC12619108; doi:10.1111/gcb.70604)
Supplement: Supplementary file 1 — Data S1: gcb70604‐sup‐0001‐DataS1.pdf. [file GCB-31-e70604-s001.pdf]

Supporting information for research article ‘Can oxygen isotopes in tree rings be used to detect stomatal responses to global change?’

Imogen Carter, Roel Brienen and Manuel Gloor

SI.1 Modelling assumptions

Appendix S1

Table SI.1: Model assumptions used to examine the effects of climate change and CO_2 on $\delta^{18}O$ between 1901 and 2023 for each case

	Péclet comparison		g_{s0} comparison		climate comparison	
Case	Without	With	Low	High	Dry	Wet
Geographical region	All land points 23-67ºN					
T_{leaf} (ºC)	20 (+ anomaly)*					
e_a (kPa)	1.4	1.4	1.4	1.4	0.94	2.1
	(+ anomaly)*					
g_{s0} (mol m^{-2} s^{-1})	0.3	0.3	0.05	0.65	0.3	0.3
Péclet effect	Without	With				

*For models 1-3, T_{leaf} and e_a are fixed at the respective values (20ºC and 1.4kPa) in Eqns. 1,3,4,7 & SI.3.1. For models 2-4, the g_s functions (Eqns. SI.3.3 & SI.3.4) use T_{leaf} = (20 + anomaly) and e_a =(1.4 + anomaly). But for models 2-3, all other points that e_a and T_{leaf} occur in the $\delta^{18}O$ equations (i.e. Eqns. 1,3,4,7 & SI.3.1) are fixed at 20ºC and 1.4kPa, respectively. For model 4, T_{leaf} = (20 + anomaly) and e_a =(1.4 + anomaly) at all points in the g_s functions and $\delta^{18}O$ equations.

Climate anomaly data downloaded from KNMI Explorer (https://climexp.knmi.nl/) is in the accompanying excel file.

SI.2 Modelling Parameters

Appendix S2

Variables that are the same in all four models and all six cases (Table 2).

Table SI.2: Other model parameters

**Parameter**	**Value**	**Unit**
Boundary layer conductance (g_b)	2.5	mol m^{-2} s^{-1}
Effective pathlength (L)	0.02	m
Biochemical fractionation of cellulose synthesis (ε_{bio})	27	‰
Source water $\delta^{18}O$	0	‰
Atmospheric vapour $\delta^{18}O$	0	‰

SI.3 Model functions

Appendix S3

Oxygen isotope models

Leaf internal vapour pressure (e_i) in kPa, assumed to be at saturation (Buck, 1996):

$$e_i = 0.61121 * exp\frac{18.678 - \frac{T_{leaf}}{234.5}}{\frac{T_{leaf}}{257.14 + T_{leaf}}} \quad [1]$$

Where T_{leaf} is in °C.

Stomatal response models

CO_2 function

$$g_s = g_{s0} + (-0.0007 * g_{s0} * \Delta CO_2) \quad [2]$$

Where g_{s0} is the average g_s at 1901 (mol m^{-2} s^{-1}) and ΔCO_2 is the change in annual CO_2 concentration (ppm) since 1901.

This is a linearisation of the modelled g_s response to CO_2 for C-3 plants from Fig. 2g in Walker *et al*. (2021).

Stewart-Jarvis functions:

Stewart-Jarvis functions return values between 0 and 1, which become coefficients to g_s (Stewart, 1988; Jarvis, 1976):

VPD:

$$f(VPD) = \begin{cases} 1, & if\ VPD < VPD_{min} \\ 1 - \frac{VPD - VPD_{min}}{VPD_{max} - VPD}, & if\ VPD_{min} < VPD < VPD_{max} \\ 0, & if\ VPD > VPD_{max} \end{cases} \quad [3]$$

Where VPD_{min} = 1kPa, and VPD_{max} = 4kPa.

Temperature:

$$f(T_{leaf}) = \begin{cases} \frac{T_{leaf} - T_{min}}{T_{opt} - T_{min}} * \left(\frac{T_{max} - T_{leaf}}{T_{max} - T_{opt}}\right)^{\frac{T_{max} - T_{opt}}{T_{opt} - T_{min}}}, & if\ T_{min} < T_{leaf} < T_{max} \\ 0, & otherwise \end{cases} \quad [4]$$

Where T_{min} = 10°C, T_{opt}= 20°C, and T_{max} = 35°C.

Although the g_s response to temperature is partially inbuilt into the g_s=f(VPD) function (as e_i is temperature-dependent), both g_s=f(VPD) and g_s=f(T_{leaf}) are required to realise the full temperature effect on g_s.

SI.4 Effects of low versus high stomatal conductance

Changes in CO_2 (climate) between 1901 and 2023 cause an 8% (10%) decrease of g_s for the low and high g_s cases (Fig. SI.4a,b- models 1(2)). In absolute terms, this reflects a reduction of 0.004 mol m^{-2} s^{-1} (0.005 mol m^{-2} s^{-1}) for the low g_s case, and 0.055 mol m^{-2} s^{-1} (0.065 mol m^{-2} s^{-1}) for the high g_s case. According to our model, total decreases in g_s due to CO_2 and climate result in an increase in $\delta^{18}O_{trc}$ between 0.09 and 0.67‰ (Fig. SI.4g,h- model 3). When the direct effects of climate are also included, $\delta^{18}O_{trc}$ increases between 0.34 and 0.69‰ (model 4).

Reductions in g_s due to CO_2 lead to negligible effects on $\delta^{18}O$ for the low g_s case because the absolute change in g_s is very small (Fig. SI.4c,e,g- model 1). In contrast, for the high g_s case, reductions in g_s due to CO_2 increase $\Delta^{18}O_{lw}$ by 0.52‰ (Fig. SI.4f- model 1). This is because transpiration flux decreases due to decreases in g_s, allowing greater back-diffusion of ^{18}O-enriched water from the evaporating site into bulk leaf water. Reductions in g_s due to climate follow similar trends to the effects described for CO_2, such that there is a negligible effect on $\delta^{18}O$ for the low g_s case (Fig. SI.4c,e,g- model 2), and $\Delta^{18}O_{lw}$ increases by 0.62‰ for the high g_s case (Fig. SI.4f- model 2).

For the low g_s case, the largest effect on $\delta^{18}O$ is the direct effect of climate, independent of changes in g_s. Warming and decreases in e_a elevate VPD, which enhances the contribution of ε_k to the isotopic signal at the site of evaporation, resulting in a 1.2‰ increase of $\Delta^{18}O_{es}$ and $\Delta^{18}O_{lw}$ (Fig. SI.4c,e,g- model 4). The direct effects of climate have the same effect on $\Delta^{18}O_{es}$ for the high g_s case (Fig. SI.4d- model 4), but this is significantly weakened in bulk leaf water, such that $\Delta^{18}O_{lw}$ only increases by 0.57‰ (Fig. SI.4f- model 4). This is because transpiration flux is much greater due to larger g_s, and thus there is a greater contribution of unenriched water from the replenishment of water loss via transpiration into bulk leaf water.

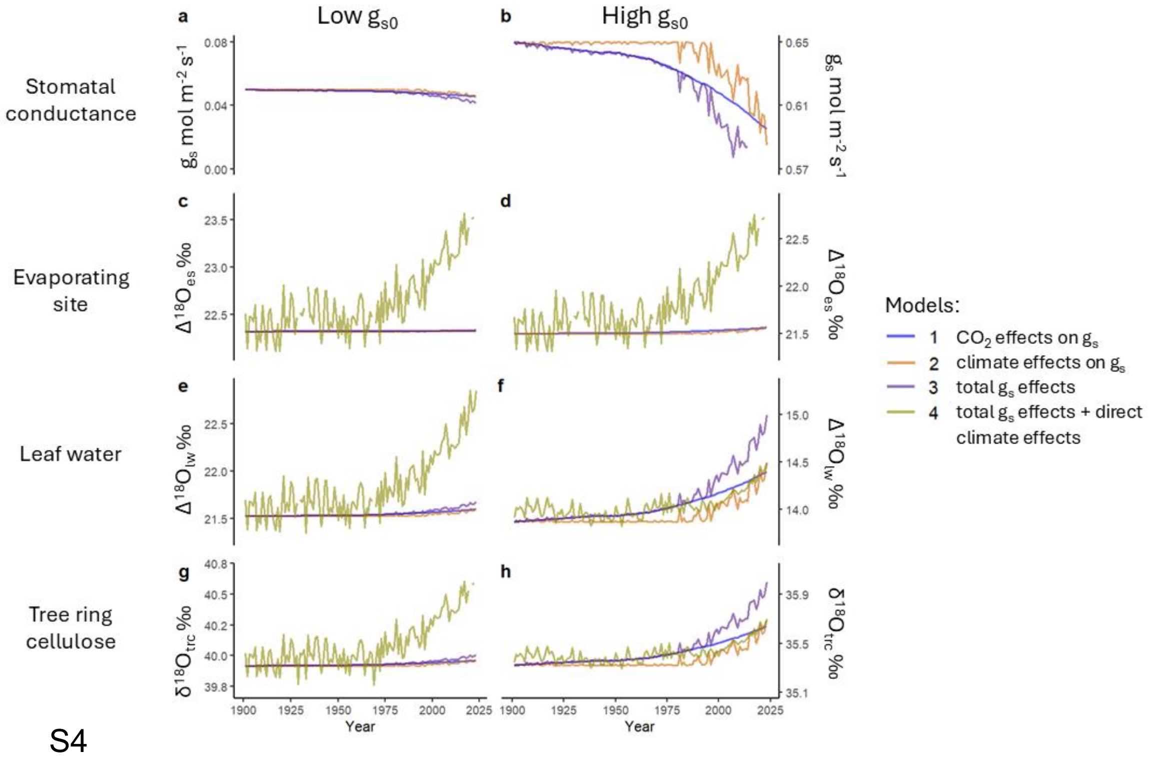

S4

Figure SI.4 Predicted $\delta^{18}O$ changes over the period 1901-2023 to changes in g_s due to CO_2 and climate, and to the direct effects of climate, for the low and high g_{s0} cases. The Péclet effect is included in all modelled responses. The top row shows the modelled g_s response to CO_2 (blue), climate (orange), and CO_2 & climate (purple) for the low g_s case (a), and for the high g_s case (b). For the low g_s case, the average g_s value in 1901 (g_{s0}) is 0.05 mol m^{-2} s^{-1}. For the high g_s case, g_{s0} is 0.65 mol m^{-2} s^{-1}. Subplots c-h show the modelled $\delta^{18}O$ response to changes in g_s and to the direct effects of climate under different levels of sensitivity (models 1-4) in $\Delta^{18}O_{es}$ (c,d), in $\Delta^{18}O_{lw}$ (e,f), and in $\delta^{18}O_{TRC}$ (g,h). 'Climate effects on g_s' refers to the modelled g_s response to VPD and temperature changes. 'Total g_s effects' refers to the modelled g_s response to changes in CO_2 plus climate. 'Direct climate effects' refers to the effects of VPD and temperature changes in the CGD model and on the rate of transpiration, independent of changes to g_s. The y-axis range for subplots c-f is 1.6‰, and is 1‰ for subplots g-h.

SI.5 Effects of dry versus wet climates

Increases in CO_2 between 1901 and 2023 cause an 8% (0.025 mol m^{-2} s^{-1}) reduction in g_s for the dry and wet climate cases (Fig. SI.5a,b- model 1). In contrast, changes in climate cause g_s to decrease by 11% in the dry climate case, but only by 3% for the wet climate case (model 2). The modelled g_s response to climate begins at a lower value for the dry climate case because VPD is already above the modelled physiological threshold (VPD_{min} c.f. SI-3-4) to elicit stomatal closure. According to our model, total decreases in g_s result in an increase of $\delta^{18}O_{trc}$ between 0.05 and 0.75‰ (Fig. SI.5g,h- model 3). When the direct effects of climate are also included, $\delta^{18}O_{trc}$ increases between 0.46 and 0.95‰ (model 4).

Reductions in g_s due to CO_2 lead to negligible effects on $\delta^{18}O$ for the wet climate case (Fig. SI.5d,f,h- model 1), but cause an increase of $\Delta^{18}O_{lw}$ by 0.55‰ for the dry climate case (Fig. SI.5e- model 1). This is because VPD is much larger in the dry climate case; therefore, decreases in g_s cause a greater decrease in leaf transpiration rate (Eqn. 7) and thus, a greater increase in the back-diffusion of ^{18}O-enriched water from the evaporating site.

Reductions in g_s due to climate follow similar trends to the effects described for CO_2, such that there is a negligible effect on $\delta^{18}O$ for the wet climate case (Fig. SI.5d,f,h- model 2), but result in an increase in $\Delta^{18}O_{lw}$ by 0.47‰ for the dry climate case (Fig. SI.5e- model 2). This is because VPD is larger and because there is a greater decrease in g_s due to climate in the dry climate case, leading to a greater decrease in transpiration rate, and thus, a greater increase in $\Delta^{18}O_{lw}$ compared to the wet climate case.

For both cases, the largest effect on $\Delta^{18}O_{es}$ is the direct effects of climate, but this effect is greater for the wet climate case (Fig. SI.5c,d- model 4). This is explained by the alternative expression of the CGD model, assuming $\delta^{18}O_{wv} = \delta^{18}O_{sw}$:

$$\Delta^{18}O_{es} \approx \varepsilon^* + \varepsilon_k(1 - \frac{e_a}{e_i})$$

There is a greater reduction in RH (e_a/e_i) in the wet (8%) versus the dry (2%) scenario, and thus, there is a greater increase in the degree of contribution of ε_k to $\Delta^{18}O_{es}$ for the wet scenario.

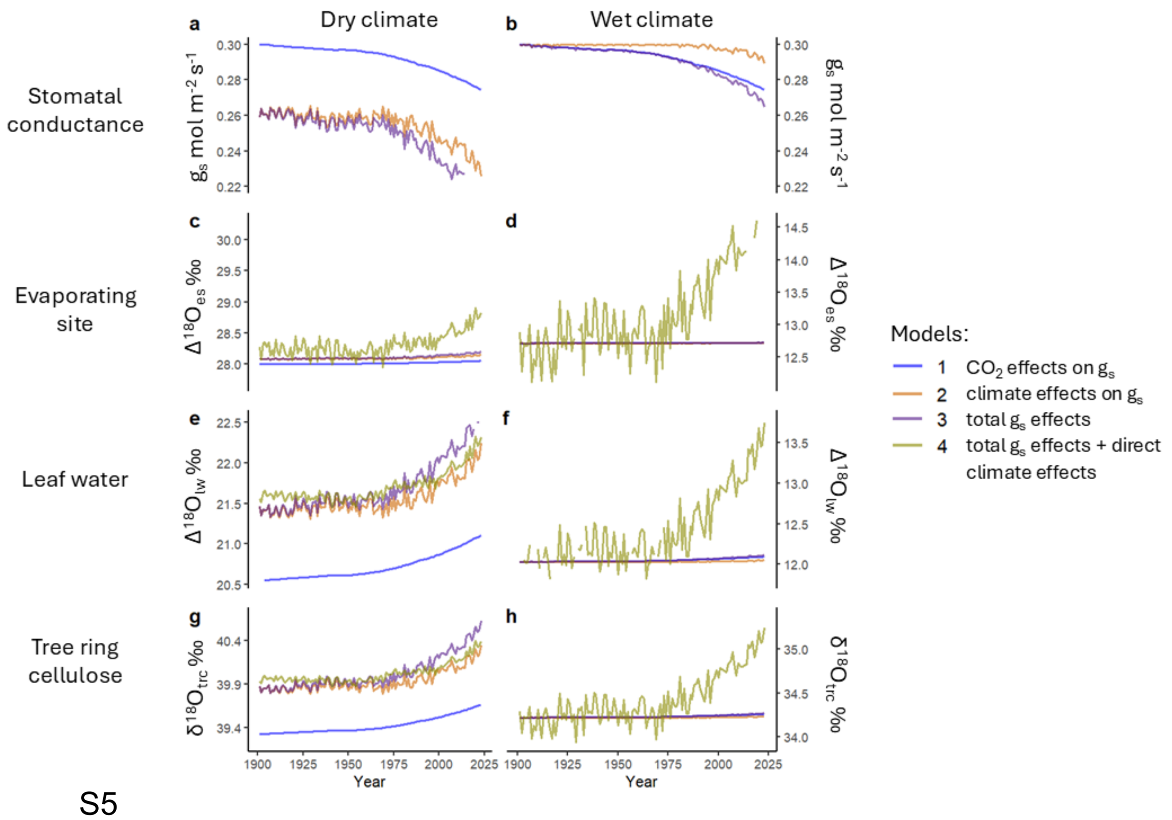

S5

Figure SI.5 $\delta^{18}O$ sensitivity to changes in g_s due to CO_2 and climate, and to the direct effects of climate, for the dry and wet climate cases. The Péclet effect is included in all modelled responses. The top row shows the modelled g_s response to CO_2 (blue), climate (orange), and CO_2 & climate (total) for the dry climate case (a), and for the wet climate case (b). For the dry climate case, the annual mean e_a in 1901 is 0.94kPa (i.e. 40% RH at 20ºC). For the wet climate case, the annual mean e_a in 1901 is 2.1kPa (i.e. 90% RH at 20ºC). Subplots c-h show the modelled $\delta^{18}O$ response to changes in g_s and to the direct effects of climate under different levels of sensitivity (models 1-4) in $\Delta^{18}O_{es}$ (c,d), in $\Delta^{18}O_{lw}$ (e,f), and in $\delta^{18}O_{trc}$ (g,h). 'Climate effects on g_s' refers to the modelled g_s response to VPD and temperature changes. 'Total g_s effects' refers to the modelled g_s response to changes in CO_2 plus climate. 'Direct climate effects' refers to the effects of VPD and temperature changes in the CGD model and on the rate of transpiration, independent of changes to g_s. The y-axis range for subplots c-d is 2.5‰, 2.0‰ for subplots e-f, and 1.5‰ for subplots g-h.

SI.6 Model performance against published tree $\delta^{18}O$ records

Here, we compare the predicted total change in $\delta^{18}O_{trc}$ by our model (model 4) with real $\delta^{18}O_{trc}$ changes for 172 chronologies collated from three studies (Guerrieri et al., 2019; Mathias & Thomas, 2023; Treydte et al., 2023). Each chronology is a unique species x site record. The most significant period of global changes over the 20th and 21st centuries have been since ~1975 (c.f. Fig. 4, main text), so we compare the last 50 years (i.e. since 1975) of $\delta^{18}O_{trc}$ changes predicted by our model, with observed 50-year changes in $\delta^{18}O_{trc}$ for each chronology (Fig. SI.6). As the $\delta^{18}O_{trc}$ records in Guerrieri et al., 2019 are only 30 years long, we compare 50-year changes for each chronology by calculating the slope (i.e. change in $\delta^{18}O_{trc}$ per year) between the start and end years given in Table SI.6, and multiplying this value by 50.

S6

Table SI.6 Summary information of published $\delta^{18}O_{trc}$ records

Study	Geographic region	Start-end year	No. of chronologies	No. of sites	No. of species
**Guerrieri et al., 2019**	North America	1982-2012	15	8	12
**Mathias & Thomas, 2021**	Global	1975-2015	113	84	36
**Treydte et al., 2023**	Europe	1975-2013	44	44	7
Note: the start-end years given are the years we use to calculate the slope (change in $\delta^{18}O_{trc}$ per year) for the last 50 years for each chronology. The full records in Mathias & Thomas, 2021 and Treydte et al., 2023 cover much longer time periods.					

There is significant variation in the 50-year $\delta^{18}O_{trc}$ changes across the 172 chronologies (Fig. SI.6), which reflects the strong influence of climatic variability and species-specific effects on $\delta^{18}O_{trc}$. The 50-year change in $\delta^{18}O_{trc}$ predicted by our model (red dashed line) is close to the mean 50-year $\delta^{18}O_{trc}$ change of the 172 published tree ring chronologies (black dashed line).

Our model assumes that $\delta^{18}O_{sw}$ does not change across time because there are no accurate long-term data of trends in $\delta^{18}O_{sw}$ used by trees, and current methods to derive temporal $\delta^{18}O_{sw}$ trends from spatial $\delta^{18}O_{sw}$ models are unreliable (Lin *et al*., 2022). However, the $\delta^{18}O_{sw}$ used by the trees in these records has likely changed over the last 50 years, to varying degrees between sites, due to increases in large-scale atmospheric temperatures (Vystavna *et al*., 2020). This $\delta^{18}O_{sw}$ variability likely also contributes to the large spread of

$\delta^{18}O_{trc}$ changes seen in Fig SI.6. Thus, the degree to which these $\delta^{18}O_{trc}$ changes reflect changes in $\delta^{18}O_{sw}$ and changes in leaf evaporative enrichment (i.e. effects of VPD and g_s changes) remains unclear.

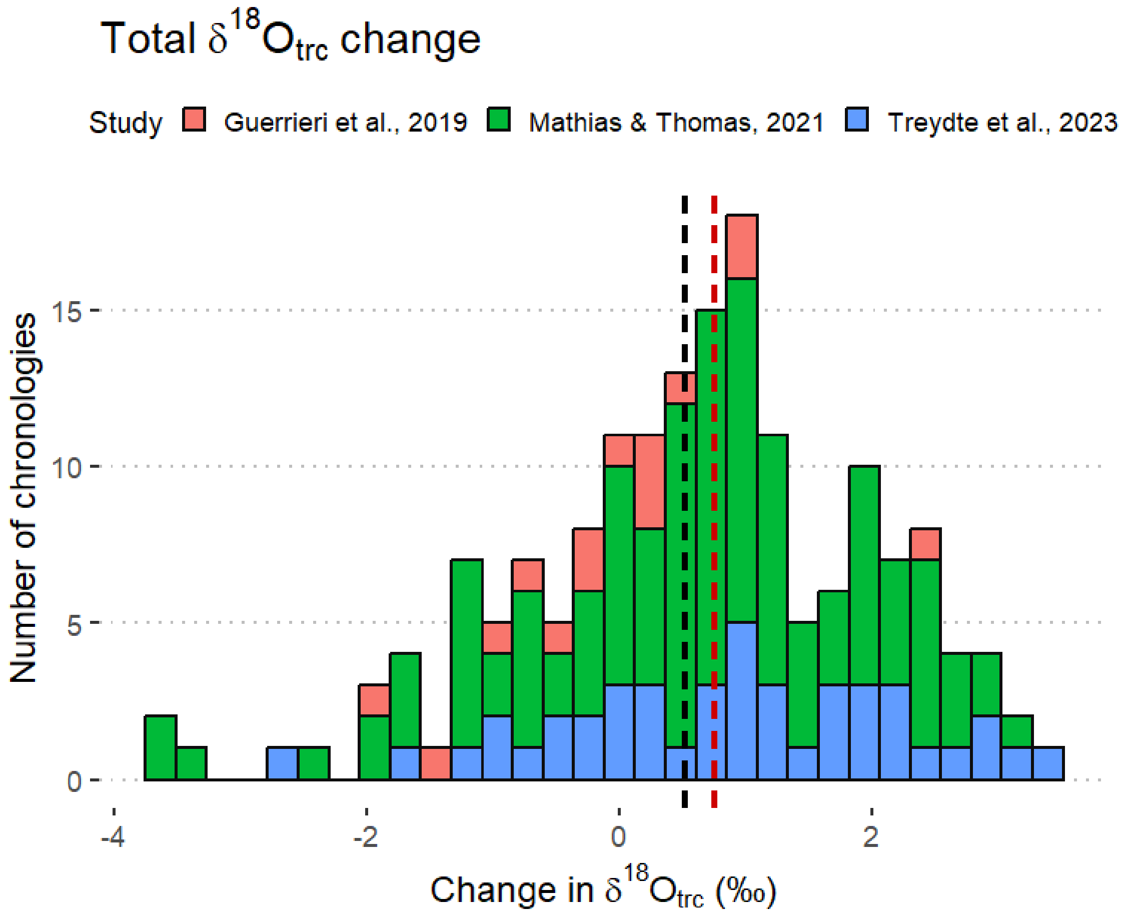

Figure SI.6 50-year change in $\delta^{18}O_{trc}$ predicted by our model compared with 50-year changes in $\delta^{18}O_{trc}$ for 172 tree ring chronologies. Red dashed line indicates the 50-year change in $\delta^{18}O_{trc}$ according to our model. Black dashed line indicates the mean 50-year change in $\delta^{18}O_{trc}$ of the 172 published chronologies.

SI.7 Model predictions for tropical climates

Here, we use the models to simulate $\delta^{18}O_{trc}$ trends between 1906 and 2023 due to changes in stomatal conductance and changes in climate, for a tropical site. Stomatal responses are modelled as given in SI.3, except the Stewart-Jarvis temperature function is adapted for tropical leaf temperatures, where T_{min} = 25ºC, T_{opt}= 32ºC, and T_{max} = 50ºC. We use the same modelling assumptions as for the temperate simulations, except leaf temperature (T_{leaf}) is 32°C plus the mean annual air temperature anomaly for land points 25ºS-20ºN, and atmospheric vapour pressure (e_a) is 3.81kPa plus the mean annual e_a anomaly for land points 25ºS-20ºN (Table SI.7). Therefore in 1906, relative humidity is 80% and VPD is 0.94kPa.

We show the simulated $\delta^{18}O_{trc}$ trends in a tropical climate for the cases with and without a Péclet effect (Fig. SI.7i), and for a low and high g_s (Fig. SI.7ii). The outcomes of these tropical simulations support our main findings from the temperate simulations, that long-term changes in g_s cannot be unambiguously disentangled from $\delta^{18}O_{trc}$ trends.

CO_2 and climate-induced decreases in g_s (Fig. SI.7i- a) do not contribute to changes in $\delta^{18}O_{trc}$ without a Peclet effect (b- models 1-3 vs 4). For the 'with Peclet effect' case, changes in g_s significantly contribute to predicted $\delta^{18}O_{trc}$ trends (c- models 3 vs 4). For the low g_s case, CO_2 and climate-induced changes in g_s do not significantly contribute to $\delta^{18}O_{trc}$ trends (Fig. SI.7ii- c- models 1-3 vs 4) because the net change in g_s is very small (a). For the high g_s case, changes in CO_2 and climate cause large decreases in g_s (b). However, these large changes in g_s are poorly reflected in the weak $\delta^{18}O_{trc}$ trend (d- models 3 vs 4).The mechanisms driving the effects of changes in g_s and climate on $\delta^{18}O_{trc}$ trends are described in the Results, SI.4 and SI.5.

Table SI.7: Model assumptions used to examine the effects of climate change and CO2 on $\delta^{18}O$ at a tropical site between 1906 and 2023 for each case

	Péclet comparison		g_{s0} comparison	
Case	Without	With	Low	High
Geographical region	All land points 25ºS-20ºN			
T_{leaf} (ºC)	32 (+ anomaly)*			
e_a (kPa)	3.81 (+ anomaly)*			
g_{s0} (mol m^{-2} s^{-1})	0.3	0.3	0.05	0.65
Péclet effect	Without	With		

*For models 1-3, T_{leaf} and e_a are fixed at the respective values (32ºC and 3.81kPa) in Eqns. 1,3,4,7 & SI.3.1. For models 2-4, the g_s functions (Eqns. SI.3.3 & SI.3.4) use T_{leaf} = (32 + anomaly) and e_a =(3.81 + anomaly). But note, for models 2-3, all other points that e_a and T_{leaf} occur in the $\delta^{18}O$ equations (i.e. Eqns. 1,3,4,7 & SI.3.1) are fixed at 32ºC and 3.81kPa, respectively. For model 4, T_{leaf} = (32 + anomaly) and e_a =(3.81 + anomaly) at all points in the g_s functions and $\delta^{18}O$ equations.

Climate anomaly data downloaded from KNMI Explorer (https://climexp.knmi.nl/) is in the accompanying excel file.

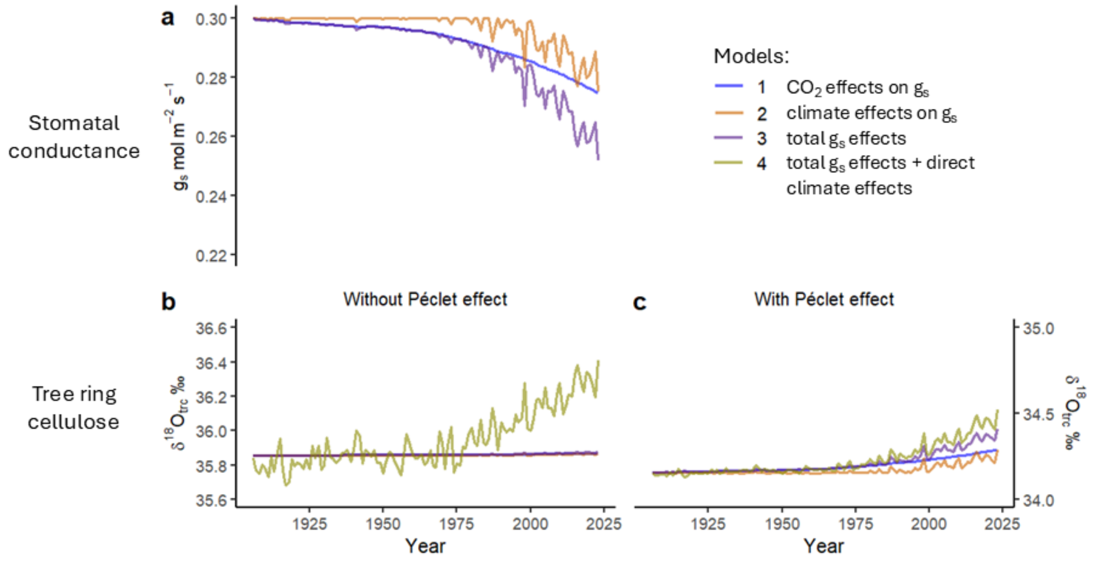

S7

Figure SI.7i Predicted $\delta^{18}O_{trc}$ changes over the period 1906-2023, with and without the Péclet effect, to changes in g_s due to CO_2 and climate, and to the direct effects of climate, for a tropical site. Subplot a shows the modelled g_s response to CO_2 (blue), climate (orange), and CO_2 & climate (purple) for both cases. Subplots b-c show the modelled $\delta^{18}O_{trc}$ response, without (b) and with the Péclet effect (c), to changes in g_s and to the direct effects of climate under different levels of sensitivity (models 1-4). Without the Péclet effect, g_s only affects $\delta^{18}O$ via ε_k (Eqn. 2), and – for model 4 only – VPD, e_a and temperature also influence $\delta^{18}O$ via ^{18}O-enrichment at the evaporating site (Eqn. 1). With the Péclet effect, g_s affects $\delta^{18}O$ via ε_k (Eqn. 2) and $\wp$ (Eqns. 6-7), and – for model 4 only – VPD, e_a and temperature also influence $\delta^{18}O$ via ^{18}O-enrichment at the evaporating site (Eqn. 1) and $\wp$ (Eqns. 6-7). The y-axis range for subplots b-c is 1‰.

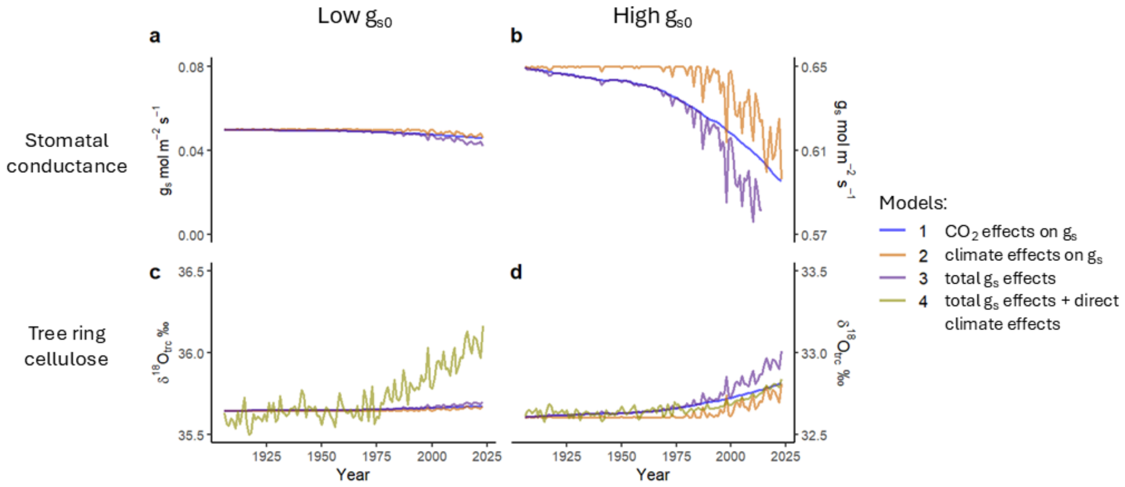

S7

Figure SI.7ii Predicted $\delta^{18}O$ changes over the period 1906-2023 to changes in g_s due to CO_2 and climate, and to the direct effects of climate, for the low and high g_s cases in a tropical climate. The Péclet effect is included in all modelled responses. The top row shows the modelled g_s response to CO_2 (blue), climate (orange), and CO_2 & climate (purple) for the low g_s case (a), and for the high g_s case (b). For the low g_s case, the average g_s value in 1901 (g_{s0}) is 0.05 mol m^{-2} s^{-1}. For the high g_s case, g_{s0} is 0.65 mol m^{-2} s^{-1}. Subplots c-d show the modelled $\delta^{18}O_{trc}$ response to changes in g_s and to the direct effects of climate under different levels of sensitivity (models 1-4). 'Climate effects on g_s' refers to the modelled g_s response to VPD and temperature changes. 'Total g_s effects' refers to the modelled g_s response to changes in CO_2 plus climate. 'Direct climate effects' refers to the effects of VPD and temperature changes in the CGD model (Eqn. 1) and on the rate of transpiration (Eqns. 6-7), independent of changes to g_s. The y-axis range for subplots c-d is 1‰.

References

Bottinga Y., Craig H. 1969. Oxygen isotope fractionation between CO2 and water, and the isotopic composition of marine atmospheric CO2. Earth and Planetary Science Letters 5, 285–295. DOI: 10.1016/S0012-821X(68)80054-8

Buck, A.L. 1996. Buck Research CR-1A User's Manual, Appendix 1.

Guerrieri, R., Belmecheri, S., Ollinger, S.V., Asbjornsen, H., Jennings, K., Xiao, J., Stocker, B.D., Martin, M., Hollinger, D.Y., Bracho-Garrillo, R., Clark, K., Dore, S., Kolb, T., Munger, J.W., Novick, K., Richardson, A.D., 2019. Disentangling the role of photosynthesis and stomatal conductance on rising forest water-use efficiency. Proc. Natl. Acad. Sci. U.S.A. 116, 16909–16914. https://doi.org/10.1073/pnas.1905912116

Jarvis, P.G., 1976. The interpretation of the variations in leaf water potential and stomatal conductance found in canopies in the field. Phil. Trans. R. Soc. Lond. B 273, 593–610. DOI: 10.1098/rstb.1976.0035

Lin, W., Barbour, M.M., Song, X., 2022. Do changes in tree-ring δ 18 O indicate changes in stomatal conductance? New Phytologist 236, 803–808. DOI: 10.1111/nph.18431

Mathias, J.M., Thomas, R.B., 2021. Global tree intrinsic water use efficiency is enhanced by increased atmospheric CO 2 and modulated by climate and plant functional types. Proc. Natl. Acad. Sci. U.S.A. 118, e2014286118. https://doi.org/10.1073/pnas.2014286118

Stewart, J.B., 1988. Modelling surface conductance of pine forest. Agricultural and Forest Meteorology 43, 19–35. DOI: 10.1016/0168-1923(88)90003-2

Treydte, K., Liu, L., Padrón, R.S. *et al.* Recent human-induced atmospheric drying across Europe unprecedented in the last 400 years. *Nat. Geosci.* **17**, 58–65 (2024). https://doi.org/10.1038/s41561-023-01335-8

Vystavna, Y., Matiatos, I. and Wassenaar, L.I. 2020. 60-year trends of δ18O in global precipitation reveal large scale hydroclimatic variations. *Global and Planetary Change*. **195**, p.103335.

Walker, A.P., *et al.* 2021. Integrating the evidence for a terrestrial carbon sink caused by increasing atmospheric CO2. New Phytologist 229, 2413–2445. DOI: 10.1111/nph.16866
